# Toward Effective and Adsorption-Based Antifouling Zipper Brushes: Effect of pH, Salt, and Polymer Design

**DOI:** 10.1021/acsapm.3c01217

**Published:** 2023-09-14

**Authors:** Anna M. C. Maan, Anton H. Hofman, Théophile Pelras, Ilan M. Ruhof, Marleen Kamperman, Wiebe M. de Vos

**Affiliations:** † Polymer Science, Zernike Institute for Advanced Materials, University of Groningen, Nijenborgh 4, 9747 AG Groningen, The Netherlands; ‡ Macromolecular Chemistry and New Polymeric Materials, Zernike Institute for Advanced Materials, University of Groningen, Nijenborgh 4, 9747 AG Groningen, The Netherlands; § Membrane Science and Technology, MESA+ Institute for Nanotechnology, University of Twente, P.O. Box 217, 7500 AE Enschede, The Netherlands

**Keywords:** antifouling coating, two-step adsorption, polymer brush, diblock copolymers, hydrophobic surfaces

## Abstract

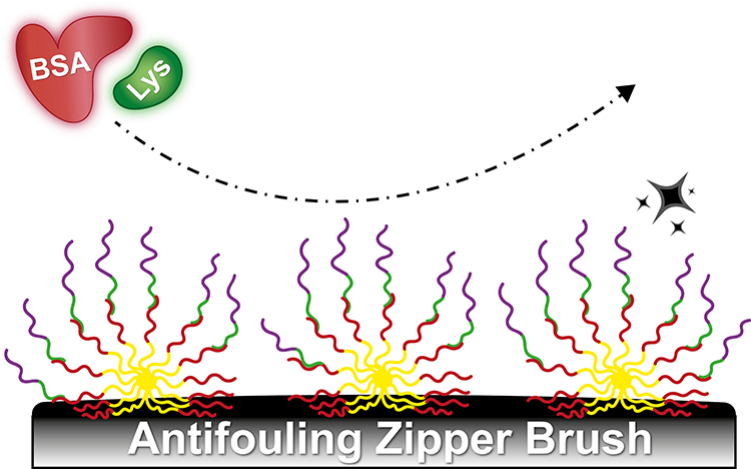

The undesired spontaneous deposition and accumulation of matter on surfaces, better known as fouling, is a problematic and often inevitable process plaguing a variety of industries. This detrimental process can be reduced or even prevented by coating surfaces with a dense layer of end-grafted polymer: a polymer brush. Producing such polymer brushes via adsorption presents a very attractive technique, as large surfaces can be coated in a quick and simple manner. Recently, we introduced a simple and scalable two-step adsorption strategy to fabricate block copolymer-based antifouling coatings on hydrophobic surfaces. This two-step approach involved the initial adsorption of hydrophobic-charged diblock copolymer micelles acting as a primer, followed by the complexation of oppositely charged-antifouling diblock copolymers to form the antifouling brush coating. Here, we significantly improve this adsorption-based zipper brush via systematic tuning of various parameters, including pH, salt concentration, and polymer design. This study reveals several key outcomes. First of all, increasing the hydrophobic/hydrophilic block ratio of the anchoring polymeric micelles (*i.e.*, decreasing the hydrophilic corona) promotes adsorption to the surface, resulting in the most densely packed, uniform, and hydrophilic primer layers. Second, around a neutral pH and at a low salt concentration (1 mM), complexation of the weak polyelectrolyte (PE) blocks results in brushes with the best antifouling efficacy. Moreover, by tuning the ratio between these PE blocks, the brush density can be increased, which is also directly correlated to the antifouling performance. Finally, switching to different antifouling blocks can increase the internal density or strengthen the bound hydration layer of the brush, leading to an additional enhancement of the antifouling properties (>99% lysozyme, 87% bovine serum albumin).

## 1 Introduction

Polluted drinking water, increasing shipping costs, and frequent occurrences of healthcare-associated infections are among the many issues primarily caused by one phenomenon: fouling.^1−3^ Fouling involves the uncontrollable adhesion and accumulation of unwanted material from the surroundings onto surfaces.^4^ Due to the many types of fouling, including organic, inorganic, and biological, all with their own size, shape, and composition, it presents an inevitable and complex challenge in many fields.^4,5^ For the maritime industry alone, the estimated cost for transport delays, hull repairs, cleaning, and general maintenance caused by biofouling is set to 150 billion dollars annually,^2^ while in the public health domain, more than 45% of the hospital-contacted infections can be attributed to biofilm-infected medical devices (*e.g.*, catheters).^6^

Polymers are promising candidates to reduce or even prevent fouling, as they are affordable, easy to process, exhibit a wide-range efficacy against an array of fouling agents, and their functionalities are readily modified to suit the application of interest.^4,7,8^ Specifically, when they are densely end-grafted to a surface, either chemically or physically, the obtained polymer brush can provide both a steric and energetic barrier to prevent fouling agents from adsorbing.^9−12^ Over the past decade, many polymer brush systems have been developed, including hydrophilic, hydrophobic, zwitterionic, and amphiphilic brushes, which have been discussed extensively in numerous reviews.^4,13−16^ However, these systems are predominantly fabricated on hydrophilic and charged surfaces, generally via covalent grafting procedures, which are not easily extended to hydrophobic surfaces. Contrary to their hydrophilic counterparts, hydrophobic substrates are less prone to chemical interactions and require more modification steps (*e.g.*, surface activation, ultraviolet (UV) cross-linking, hydrosilylation) before a dense and stable coating can be produced.^17−20^ The lack of efficient solutions toward protecting said surfaces leads to significant complications, as they are essential for many fouling-prone applications within the medical sector (*e.g.*, catheters, vascular grafts, prosthetics, and implants),^13,19,21,22^ maritime transport (*e.g.*, painted ship hulls),^23^ and industry (*e.g.*, pipelines and packaging).^24^ Furthermore, the inevitable fouling process limits the lifetime of any antifouling coating, which in the case of covalently grafted coatings necessitates the use of expensive and environmentally unfriendly cleaning protocols.^25−28^

Efforts to overcome these substrate-specific and nonrenewable issues led to the development of the “zipper brush” approach.^29,30^ In this strategy, renewable and dense antifouling brushes were generated on hydrophobic surfaces by complexing diblock copolymers comprising a charged anchoring block and a neutral antifouling block to a preadsorbed and oppositely charged polyelectrolyte (PE) brush. The grafting density of the formed neutral “zipper brushes” could be controlled by tuning the chain length and grafting density of the PE brush as well as by the chain length of the charged anchoring block. Moreover, considering the electrostatic nature underlying the formation of these brushes, the complexed brush could be disintegrated and released by simply adding salt or by varying the pH, thereby restoring the original PE brush, which could subsequently be recoated in a similar fashion. One major downside accompanying this promising approach is the use of a time-consuming and scale-limiting Langmuir–Blodgett (LB) technique, which prevents its translation to large-scale applications.^29,30^

To build on this work, we recently introduced a scalable two-step adsorption strategy to fabricate zipper brushes on polystyrene surfaces.^31^ In this approach, we exchanged the scale-limiting LB-attached PE brush with an adsorbed layer of diblock copolymer micelles, consisting of a hydrophobic core and a weak PE corona (*i.e.*, the primer). This negatively charged primer was subsequently complexed with another diblock copolymer, comprising an oppositely charged weak PE block and a neutral hydrophilic block, to form an antifouling polymer brush. While these adsorption-based zipper brushes managed to effectively suppress the attachment of positively charged lysozyme, they could not prevent the adhesion of negatively charged bovine serum albumin (BSA). Hence, albeit a highly promising strategy, foulants could still adhere to the adsorbed brush, which can occur in three ways: (i) adsorption on top of the brush; (ii) adsorption within the brush; and (iii) penetration through the brush and adsorption onto the substrate.^11^ Hence, by minimizing the distance between tethered chains (high grafting density), increasing the distance between the substrate and foulant (sufficient brush thickness), eliminating any electrostatic interactions (charge neutrality), and/or strengthening the bound hydration layer (hydrophilicity), these modes of adsorption can be reduced. Many parameters such as the pH, salt concentration, and polymer design can considerably affect the aforementioned brush characteristics and, therefore, present the perfect tool to tune the antifouling properties.

**Scheme 1:**
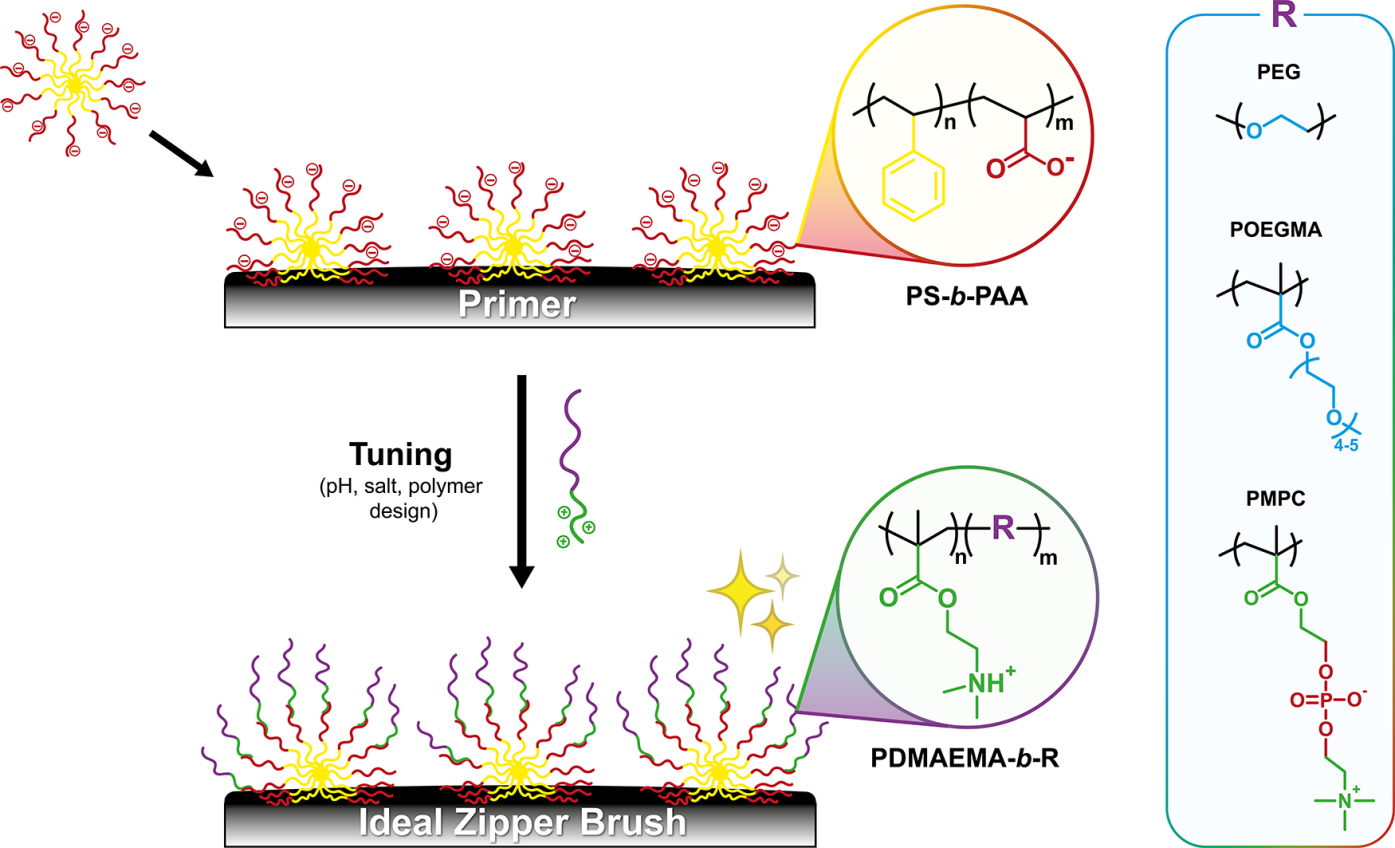
Schematic Representation of the Adsorption-Based Zipper Brush Obtained via a Two-Step Adsorption Strategy That Involves the Initial Adsorption of Negatively Charged PS-*b*-PAA Micelles, Followed by the Complexation of Oppositely Charged and Antifouling PDMAEMA-*b*-R Diblock Copolymers (R = PEG, POEGMA, or PMPC) The zipper brush characteristics (*i.e.*, grafting density, thickness, wettability, and charge) can be optimized by tuning various parameters, including pH, salt concentration, polymer block ratios, and the nature of the antifouling block.

Here, we demonstrate a significant enhancement of the antifouling efficacy of the previously established adsorption-based zipper brush by investigating these parameters (Scheme 1). The effect of each of these parameters on the adsorption kinetics, surface topography, thickness, grafting density, and wettability of the resulting primer layers and zipper brushes, as well as the antifouling capability against two fouling agents (*i.e.*, lysozyme and BSA), was investigated using a combination of techniques, including quartz crystal microbalance with dissipation (QCM-D), atomic force microscopy (AFM), ellipsometry, and contact angle (CA) measurements.

## 2 Experimental Section

### 2.1 Materials

Monopotassium phosphate (KH_2_PO_4_, ≥99.0%), bovine serum albumin (BSA, lyophilized powder, 66 kDa, ≥96%), lysozyme (Lys, lyophilized powder from chicken egg white, 14.3 kDa, ∼100,000 U mg^–1^), and hydrogen peroxide (30% solution) were purchased from Sigma-Aldrich. Sodium hydroxide (NaOH) pellets were obtained from Acros Organics. Hydrochloric acid (37–38% solution) and ammonia (25% solution, for analysis) were acquired from Boom. Absolute ethanol (99.9%) was purchased from J.T. Baker. QCM-D sensors with a gold coating (QSense, QSX 301 Gold) were purchased from Quantum Design GmbH, Germany. Milli-Q water was obtained from a Labconco WaterPro PS system in which water is purified four times: carbon filters, 2× deionization, and organic adsorption. All chemicals were used as received.

### 2.2 Polymer Synthesis

A collection of diblock copolymers with varying block ratios and lengths was successfully synthesized through controlled radical polymerization protocols. The required materials, detailed experimental procedures, and extensive analyses of each copolymer can be found in the Supporting Information (SI–Section 1). Polystyrene-*block*-poly(acrylic acid) (PS-*b*-PAA) (Tables S1 and S2 and Figures S1–S4), poly(2-(dimethylamino)ethyl methacrylate)-*block*-poly(oligo(ethylene glycol) methyl ether methacrylate) (PDMAEMA-*b*-POEGMA) (Figures S5–S7), and poly(2-(dimethylamino)ethyl methacrylate)-*block*-poly(2-methacryloyloxyethyl phosphorylcholine) (PDMAEMA-*b*-PMPC) (Figure S8) were obtained via reversible addition–fragmentation chain transfer (RAFT) polymerization. Poly(2-(dimethylamino)ethyl methacrylate)-*block*-poly(ethylene glycol) (PDMAEMA-*b*-PEG) (Tables S3 and S4 and Figures S9 and S10) was synthesized through atom transfer radical polymerization (ATRP). Table 1 provides an overview of the utilized copolymers. Within the performed experiments, the nearly identical PS_81_-*b*-PAA_81_, PS_81_-*b*-PAA_79_, and PS_85_-*b*-PAA_81_ copolymers were used interchangeably and are collectively referred to as PS_81_-*b*-PAA_81_.

**Table 1:** Overview of the Utilized Diblock Copolymers^a^

diblock copolymer	*M_n_* (kg mol^–1^)	*Đ*	ρ (g cm^–3^)
PS_32_-*b*-PAA_100_	10.9	1.27^b^	1.29
PS_27_-*b*-PAA_287_	23.9	1.20^b^	1.36
PS_27_-*b*-PAA_436_	34.6	1.79^b^	1.38
PS_81_-*b*-PAA_81_	14.6	1.15^b^	1.19
PDMAEMA_30_-*b*-POEGMA_97_	34.0	1.28	1.17
PDMAEMA_30_-*b*-PMPC_106_	36.2	ND^c^	1.30
PDMAEMA_29_-*b*-PEG_90_	8.7	1.22	1.22
PDMAEMA_54_-*b*-PEG_90_	12.5	1.25	1.25
PDMAEMA_84_-*b*-PEG_90_	17.3	1.27	1.27
PDMAEMA_114_-*b*-PEG_90_	22.1	1.25	1.28

a The subscripts denote the degree of polymerization of each block. The molecular weights (*M_n_*) were determined by ^1^H NMR, based on initial concentrations and calculated conversions. The molecular weight distributions (*i.e.*, dispersities) (*Đ*) were determined by GPC. The average polymer densities (ρ) were calculated by taking the weight average of the density of each block of the corresponding copolymer (SI – Section 2).

b Due to undesired interactions with the GPC column, these dispersities were measured only for the corresponding protected PS-*b*-P*t*BA precursors.

c The poor solubility of PMPC in organic eluent prevented the GPC measurement of PDMAEMA-*b*-PMPC in DMF (LiBr), but the conversion was deliberately kept low (∼61%) to avoid chain–chain coupling.

### 2.3 Sample Preparation

#### 2.3.1 Preparation of Phosphate Buffers

Phosphate buffers of varying ionic strength and pH were prepared by the dissolution of monopotassium phosphate (KH_2_PO_4_) and sodium hydroxide (NaOH) in Milli-Q water. For optimization of the salt concentration, 500 mL buffer stocks of pH 7 were prepared with varying ionic strengths: 1 mM (0.068 g of KH_2_PO_4_, 0.012 g of NaOH), 10 mM (0.68 g of KH_2_PO_4_, 0.12 g of NaOH), 100 mM (6.81 g of KH_2_PO_4_, 1.19 g of NaOH), and 1 M (68.1 g of KH_2_PO_4_, 11.9 g of NaOH). For optimization of the pH, 500 mL buffer stocks of constant ionic strength (10 mM), but with varying pH were prepared by only changing the amount of NaOH added: pH 6 (0.023 g of NaOH), pH 7 (0.12 g of NaOH), pH 7.5 (0.17 g of NaOH), and pH 8 (0.19 g of NaOH). The buffer solutions were filtered before use (grade 15 filter paper) if required.

#### 2.3.2 Preparation of Polymer and Fouling Solutions

All diblock copolymer and fouling solutions (BSA and lysozyme) were prepared in phosphate buffer at concentrations of 1.0 mg mL^–1^, 1 day prior to the QCM-D measurements, except for PS-*b*-PAA: these copolymers were dispersed in absolute ethanol, 1 week before measuring, to allow the self-assembling system to reach an equilibrium state. All solutions were placed in a shaker to facilitate dissolution. Each solution was filtered before use (grade 15 filter paper) if required.

#### 2.3.3 Substrate Cleaning

The AT-cut gold-plated QCM-D quartz crystal sensors (QSX 301 Gold, Quantum Design, Germany) with a diameter of 14 mm were thoroughly cleaned according to a slightly modified protocol provided by QSense: sonication and rinsing in toluene (15 min); drying with N_2_; base piranha etching in a 5:1:1 mixture (v/v) of Milli-Q/NH_3_ (25%)/H_2_O_2_ (30%) at 75 °C (15 min); cooling in piranha solution (10 min); rinsing with Milli-Q water; drying with N_2_; UV/ozone treatment (10 min); and immediate spin-coating of polystyrene. The first step was included to remove the previously spin-coated PS thin film and other adsorbed polymers, which was essential for a proper recycling of the sensors.

#### 2.3.4 Substrate Modification

The clean QCM-D sensors were modified with a PS thin film by spin-coating a 0.45-μm-filtered solution of 1.5 wt % PS (*M_n_* = 44.5 kg mol^–1^, *Đ* = 1.03) in toluene at 4000 rpm for 60 s. To promote its adhesion to the surface, the PS thin films were thermally annealed in an oven for 20 min at 120 °C, after which the dry thickness was determined by ellipsometry (41.2 ± 1.1 nm).

### 2.4 Characterization

#### 2.4.1 pH Measurement

The pH of all aqueous solutions (*i.e.*, buffer, polymer, and fouling solutions) was measured using a pH meter (Mettler Toledo FiveEasy FP20, LE438 electrode). In the case of deviating pH values, NaOH (0.1 and/or 1.0 M) and HCl (0.1 and/or 1.0 M) solutions were added in small quantities until the desired pH was reached.

#### 2.4.2 Dynamic Light Scattering (DLS)

The hydrodynamic diameter and polydispersity of the PS-*b*-PAA micelles were determined via DLS on a Malvern Panalytical Zetasizer Ultra system equipped with an avalanche photodiode detector (APD) and a He–Ne laser (λ = 633 nm). Unless stated otherwise, all polymer solutions were prepared in ethanol with a concentration of 1.0 mg mL^–1^ and measured inside a 10 mm × 10 mm quartz cuvette without prior filtering. Samples were recorded in 5-fold in the noninvasive backscattering (NIBS) mode (173° detector angle) at 25 °C after a 120 s equilibration time. Results were analyzed using ZS Xplorer software (version 3.1). The reported hydrodynamic diameters and standard deviations are the averaged values based on these five consecutive measurements, while taking into account the precision and accuracy of the device (±2%).

#### 2.4.3 Quartz Crystal Microbalance with Dissipation (QCM-D)

The adsorption and complexation of polymers, as well as the response of the adsorbed layers to fouling solutions, were monitored *in situ* using a four-channel QSense E4 system connected to a peristaltic pump (Ismatec IPC). The thoroughly cleaned and PS-coated QCM-D sensors were inserted in the QCM-D flow modules (QFM 401), which were connected in parallel inside the analyzer. All QCM-D measurements were conducted at a constant flow rate of 150 μL min^–1^ and at a temperature of 22 °C. Prior to each measurement, the sensors were sequentially stabilized in air (1 h) and ethanol (1 h) to minimize any noise and/or drift. Once stabilization was reached, the measurement was started, involving cycled switching of solutions: (1) baseline in ethanol (20 min), polymer 1 (50 min), rinsing in ethanol (30 min); (2) solvent switch to phosphate buffer (20 min), polymer 2 (50–60 min), rinsing in buffer (30 min); and (3–optional) BSA or lysozyme (40 min), rinsing in buffer (30 min). The obtained polymer coatings were removed from the flow modules and gently dried with N_2_, after which the dry layer characteristics were determined with AFM, VASE, and CA. The wet thickness of the polymer coatings (*i.e.*, including bound water) was estimated by employing the viscoelastic Voigt model in Qtools (version 3.1). However, the complicated multilayer design of the coatings necessitated the use of several assumptions within the fitting procedure (*e.g.*, no intercalation between individual layers), and so the quoted values should be considered rough estimates. Nevertheless, the data do give a valuable indication regarding the hydration of the films. With regard to assessing the antifouling performance (*i.e.*, adsorption of model proteins), it was decided to compare the QCM-D data on a qualitative basis, rather than quantitatively, for two main reasons. First, in case model proteins adsorb, they do not necessarily form a neat layer on top; they may also adsorb within the brushy structure. Second, the dynamic and nonhomogeneous character of the multilayer coatings hampered a reliable modeling of the fouled “layer”.

The obtained QCM-D data include the frequency response (Δ*f*) related to the mass adsorbed, as well as the change in energy dissipation (Δ*D*) representing the viscoelasticity of the adsorbed layer. A dense or rigid layer is characterized by a small dissipation of energy, while a layer with a looser or more flexible structure exhibits a larger dissipation. The QCM-D response was monitored at the fundamental resonance frequency (4.95 MHz, *n* = 1) and at higher overtones (*n* = 3, 5, 7, 9, 11, 13). Due to insufficient energy trapping, the Δ*f* and Δ*D* values from the fundamental frequency were usually noisy and were therefore excluded from further analysis. Unless mentioned otherwise, for the purpose of clarity, all included QCM-D graphs contain only the fifth harmonic overtone (*n* = 5), depicting the total shifts in frequency (F5, blue) and energy dissipation (D5, red). In addition, with regard to the graphs depicting the complexation step (2) and antifouling performance (3), which were measured sequentially in time, the starting frequencies and dissipations were corrected to zero in order to more clearly illustrate the influence of changing parameters (pH, salt, and polymer design).

To confirm the reproducibility of the data, all experiments were performed at least twice. However, regarding the optimization of the pH and the PS/PAA block ratio, the respective experiments were performed just once using the conditions stated but have been measured more frequently at deviating solvent conditions.

#### 2.4.4 Atomic Force Microscopy (AFM)

The surface topography and roughness of the dry coated surfaces were determined using a Bruker Icon AFM system, operating at room temperature using the standard tapping mode in air. Images were recorded using a Bruker VTESPA-300 cantilever with a nominal tip radius of 5 nm and a force constant of 42 N m^–1^. Height and phase images with varying scan sizes (0.5 × 0.5 μm^2^ to 5 × 5 μm^2^) were obtained using a 0.5 or 1.0 Hz scan rate and 256 samples/line (*i.e.*, a 256 × 256 pixel resolution). For each scan size, images were recorded on at least three spots on the coated sensor surface. The obtained raw images were processed using Gwyddion software (version 2.55), employing a second-order polynomial algorithm to flatten the data. The corrected images were subsequently analyzed within the same software to determine the average root-mean-square (RMS) surface roughness (*S*_q_) and the corresponding standard deviation of each coating, based on at least six AFM height images with varying scan sizes, while taking into account the precision and accuracy of the machine (±1 Å).

#### 2.4.5 Variable-Angle Spectroscopic Ellipsometry (VASE)

The dry thickness of the coatings was recorded in air by using a calibrated JAW V-VASE ellipsometer (J.A. Woollam Co., Inc.) operating at room temperature. The measurements were performed in the spectral range of λ = 300–1700 nm and at two different angles of incidence (70 and 75°) with respect to the surface normal. The thickness was calculated by fitting the obtained ellipsometric angles (ψ, Δ) in the supplied WVASE32 software by using a three-layer model: Au/Cauchy (PS)/coupled Cauchy (primer or brush), with parameters *A_n_* = fitted (PS = 1.54, primer/brush = coupled), *B_n_* = 0.01, *C_n_* = 0, and *k* = 0. The models were fitted using the specified spectral range λ = 600–1700 nm. Each coating was measured two times on different spots, so the reported values indicate the average dry thickness ± the standard deviation, while taking into account the precision and accuracy of the instrument (±0.1%).

#### 2.4.6 Contact Angle (CA) Measurement

The wettability of the dry coatings was investigated using a Dataphysics OCA 15EC optical contact angle-measuring device equipped with an automated microsyringe and a camera. Under ambient conditions, sessile droplets of Milli-Q water with a volume of 2 μL were dispensed and gently placed on top of the coated surface, after which a snapshot was immediately taken. The static contact angles were subsequently calculated using SCA20_U software. The measurement was repeated three times at different positions on the coated surface. The presented numbers are thus averaged values ± standard deviation.

#### 2.4.7 Streaming Potential Measurement

To determine the surface zeta potential (ζ) of each coating, streaming potential measurements were conducted on a SurPASS Electrokinetic Analyzer (Anton Paar, Germany) at a constant temperature of 22 °C. Duplicate samples of each coating were first produced within QCM-D on PS-coated sensors, after which they were immediately fixed inside an adjustable gap cell using poly(phenylene sulfide) (PPS) disk-shaped sample holders (*d* = 14 mm), separated by a 100 μm spacer foil. Once inserted in the machine, the cell was rinsed several times with a 1 mM KCl electrolyte solution (pH 7) and the gap was adjusted to 140 μm. After ensuring linear flow at a pressure of 200 mbar, the surface ζ-potential was first measured at pH 7, followed by a pH sweep from 6 to 9. Every measurement included four ramps. The measuring cell was thoroughly rinsed with Milli-Q between each experiment until the recorded conductivity was well below 0.1 mV. As a reference, streaming potentials were also recorded on the pristine gold-plated sensor, PS-coated sensor, and the intermediate PS-*b*-PAA primer. The obtained data was further analyzed using Attract software (version 2.1).

## 3 Results and Discussion

### 3.1 Coating Formation

**Figure 1:**
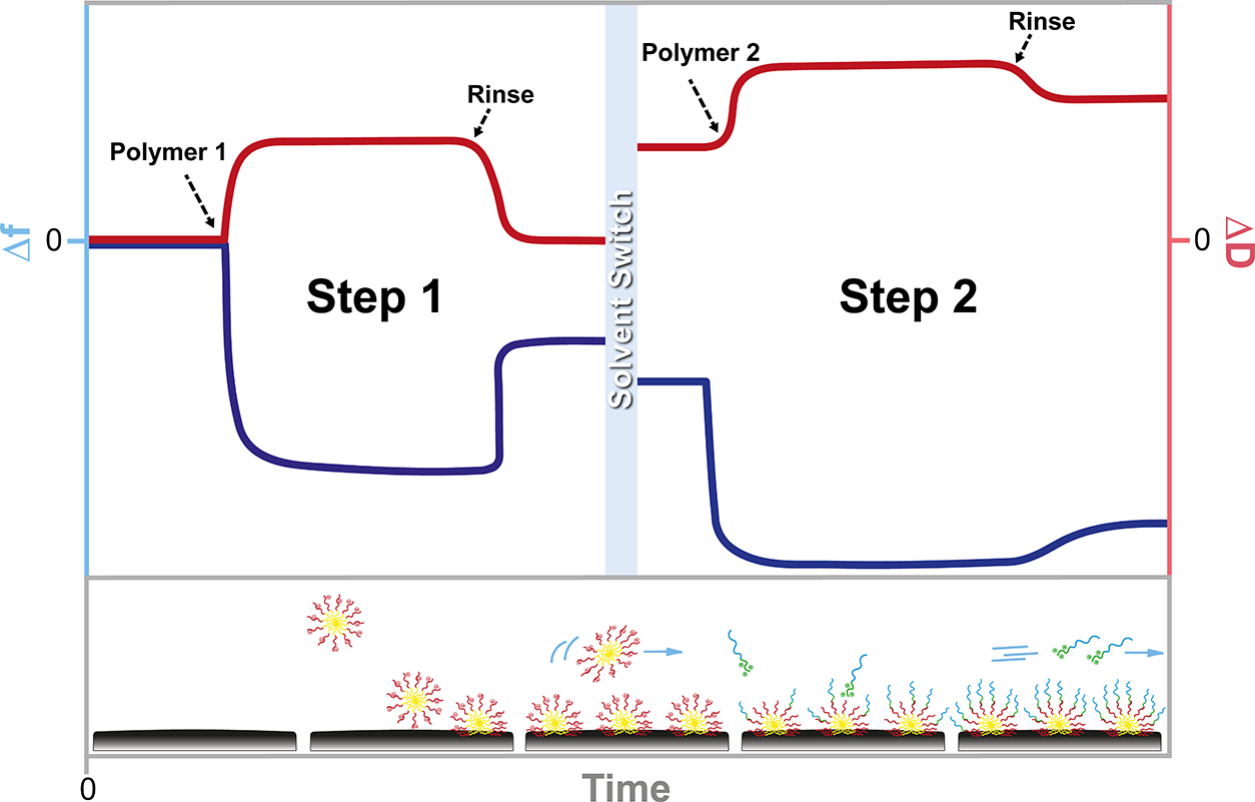
Schematic QCM-D graph illustrating the adsorption kinetics of the previously established adsorption-based zipper brush, involving the successive adsorption of PS-*b*-PAA micelles (step 1) and PDMAEMA-*b*-PEG diblock copolymer (step 2). QCM-D records the frequency response (Δ*f*, blue) related to the mass adsorbed, as well as the change in energy dissipation (Δ*D*, red) representing the rigidity of the formed polymer coating. Here, the kinetics of both adsorption steps can be described by a fast adsorption regime, an equilibrium regime, and subsequent removal of weakly attached material during rinsing.

The adsorption kinetics of the previously established adsorption-based zipper brush are most easily explained via a schematic QCM-D graph (Figure 1).^31^ In QCM-D, two parameters are simultaneously recorded: the frequency response (Δ*f*), which is related to the mass adsorbed, and the change in energy dissipation (Δ*D*), representing the rigidity of the adsorbed layer. When the dissipation is (close to) zero, the adsorbed polymer film is relatively rigid, but when the dissipation increases, it indicates the formation of a more viscous and hydrated layer.

Prior to the two-step adsorption protocol, the gold QCM-D sensors were rendered hydrophobic by spin-coating a polystyrene thin film on top (∼40 nm). The PS-*b*-PAA diblock copolymers were dispersed in ethanol, a selective solvent for PAA, which facilitated their self-assembly into micelles, consisting of a hydrophobic PS core and a negatively charged PAA corona, as evidenced by DLS (Figure S11 and Table S5). The first step involves the adhesion of these self-assembled PS-*b*-PAA micelles, which is marked by three distinct regimes (Figure 1, step 1): (i) an initial rapid adsorption of micelles to the PS-coated sensor (Δ*f* < 0), followed by (ii) an adsorption equilibrium (plateau) as the approaching micelles have to overcome an osmotic barrier generated by formerly adsorbed micelles (Δ*f* = 0), and finally (iii) mass loss of loosely attached or unbound micelles when rinsed with the reference solution (Δ*f* > 0). While undetectable in QCM-D, it is assumed that micelle adsorption involves initial deformation of the corona, which is necessary to bring the hydrophobic core into contact with the surface to form a strong bond; a phenomenon that has been observed before.^32,33^ During rinsing, the dissipation moves back to zero, suggesting the formation of a rigid PS-*b*-PAA primer. Before the second adsorption step, the reference solution is switched to buffer. The resulting frequency and dissipation shifts are predominantly related to a change in medium viscosity and density, but it also marks the slight hydration of the primer by bound water (*i.e.*, dynamic mass).^34^ Once a stable baseline is achieved, the PDMAEMA-*b*-PEG copolymer is added to the system (Figure 1, step 2). The kinetics of this complexation step can once again be described by a fast adsorption regime, an equilibrium regime, and subsequent removal of weakly complexed material. The final rinsing step is generally characterized by a minor frequency change, which implies a strong interaction between the two complexed polymer layers. At low salt concentrations, the complexation step is defined by a positive dissipation shift, emphasizing the viscous and hydrated nature of the final zipper brush. Yet, at higher salt concentrations, a negative dissipation shift is often observed, which could be explained by a loss of flexibility and/or the release of bound counterions and water molecules when the PDMAEMA-*b*-PEG copolymers penetrate and complex to the stretched out PAA chains.^35^

For reference, a complete QCM-D data set including the adsorption, complexation, and antifouling steps of the most well-established PEG-based zipper brush can be found in the SI (Figure S12). However, for the purpose of clarity, all QCM-D graphs included in the main text only consider the fifth harmonic overtone.

### 3.2 pH

The zipper brush formation is complete after complexation between two weak PE blocks: the negatively charged PAA block (p*K*_a_ = 4.5)^36^ of the primer and the oppositely charged PDMAEMA block (p*K*_a_ = 7.8)^37^ of the complexing copolymer. While strong polyelectrolytes have a fixed degree of dissociation and are essentially always charged, the net charge of these two weak polyelectrolytes is strongly determined by the pH.^7,38^ Hence, the pH must be carefully tuned in order to maximize the charge density on both blocks, thereby strengthening the complex and potentially enabling the formation of a charge-neutral brush via full charge compensation. Here, it is important to be aware of charge regulation: when two weakly charged polyelectrolytes complex, they may mutually induce further charging.^39,40^

**Figure 2:**
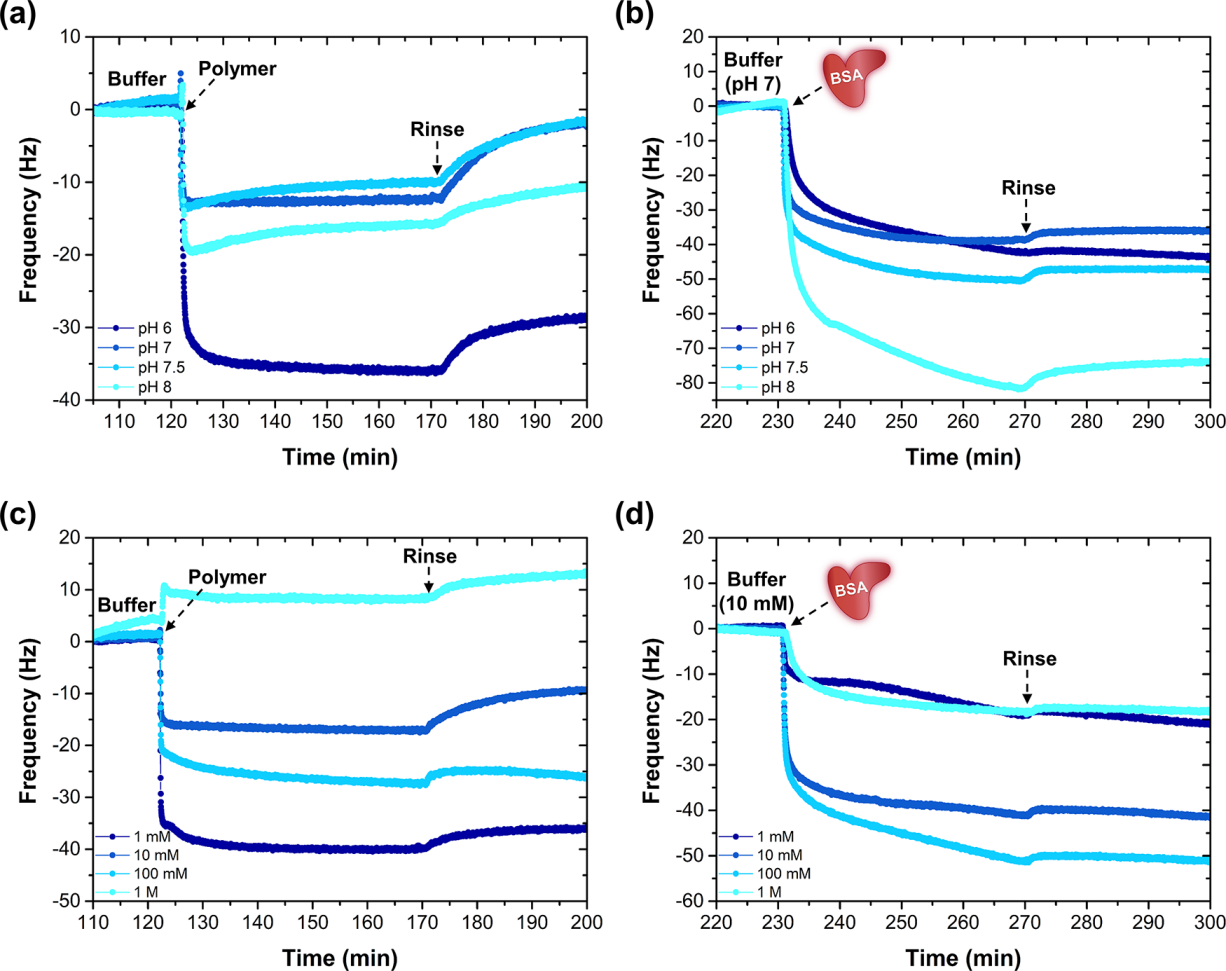
QCM-D graphs showing the effect of (a, b) pH and (c, d) ionic strength on the complexation behavior and antifouling performance of the formed zipper brush coatings. The complexation of PDMAEMA_29_-*b*-PEG_90_ diblock copolymers to preadsorbed PS_81_-*b*-PAA_81_ was followed *in situ* in (a) 10 mM phosphate buffers of varying pH and in (c) pH 7 phosphate buffers of varying ionic strength. (b, d) Antifouling performance of the obtained zipper brushes, tested against BSA at a constant pH and ionic strength (pH 7, 10 mM).

To investigate the effect of pH on the complexation behavior and antifouling performance of the adsorbed zipper brushes, the two-step adsorption procedure was performed in phosphate buffers of varying pH (pH 6 to 8), but with a constant salt concentration (10 mM) and by using only one combination of diblock copolymers (PS_81_-*b*-PAA_81_ with PDMAEMA_29_-*b*-PEG_90_). The minimum pH was set at pH 6, as both PE blocks should have a similar degree of dissociation at this pH, based on their dissociation constants.^30^ According to the obtained QCM-D data (Figure 2a), complexation between the PE blocks is most efficient at pH 6, as evidenced by the significant net frequency shift. At this pH, both blocks are sufficiently charged, which explains the substantial complexation driving force seen. At a higher pH, the electrostatic interaction seems weakened, indicated by a minimized decrease in the frequency shift.

Due to the pH dependence of charged BSA (pI 4.5),^41^ it was decided to first equilibrate all brushes in pH 7 buffer after which the antifouling performance was tested and assessed. Interestingly, the efficiency of complexation does not necessarily dictate its antifouling performance (Figure 2b): adhesion of negatively charged BSA is minimized for the zipper brush formed at pH 7. However, one has to keep in mind that a change in pH can affect many polymer properties, including the charge density, solubility, chain flexibility, and conformation. All of these factors will weigh in when complexing to the primer and will determine its antifouling efficacy. For instance, a too high adsorption of PDMAEMA-*b*-PEG copolymer at pH 6 could lead to an excess of positive charge in the final coating, which subsequently promotes BSA adsorption.

Since the adsorbed zipper brush obtained at pH 7 possessed the best antifouling efficacy, all successive zipper brushes were produced at this optimal pH.

### 3.3 Salt Concentration

Salt concentration presents another parameter that determines the strength of complexation between PAA and PDMAEMA. While salt ions are essential to facilitate proper dissociation of the respective weak polyelectrolytes, the driving force for their complexation decreases at higher ionic strengths, as the entropy gain upon the release of their counterions is reduced.^7,42^ Hence, the ionic strength of the dissolving medium must be carefully selected.

To investigate the effect of salt on the complexation behavior and antifouling performance of the adsorbed zipper brushes, the two-step adsorption procedure was performed in phosphate buffers of varying ionic strength (1 mM to 1 M), but with a constant and optimized pH of 7 and by using only one combination of diblock copolymers (PS_81_-*b*-PAA_81_ with PDMAEMA_29_-*b*-PEG_90_). According to the QCM-D data, complexation appears to be most efficient at the lowest ionic strength of 1 mM, as was expected (Figure 2c). At the highest salt concentration of 1 M, complexation is completely inhibited by a significant screening of charges, preventing the proper formation of a zipper brush.^42^

Due to an increased screening of the negatively charged BSA at higher ionic strengths, it was decided to first equilibrate all brushes in 10 mM buffer, after which the antifouling performance was tested and assessed. Interestingly, the efficiency of complexation also translates to the antifouling performance of the resulting coatings (Figure 2d): zipper brushes formed at lower ionic strengths can more effectively suppress the adhesion of BSA. Surprisingly, even though complexation seemed hindered at a 1 M salt concentration, BSA was not completely repelled by the remaining like-charged primer. This phenomenon has been studied before by de Vos et al., in which they concluded that the main driving force can be attributed to charge regulation, suggesting that the protein can reverse its charge in the vicinity of a like-charged brush.^43^

Hence, the best-performing zipper brushes are produced at neutral pH and at a low ionic strength of 1 mM.

### 3.4 Block Ratio and Block Length

Molecular weight and composition represent two other parameters that allow control over the final grafting density and thickness of the brush. In this section, we study how a change in block ratio or length can affect the adsorption kinetics, surface topography, thickness, and wettability of the resulting primer layers and zipper brushes. For the sake of clarity, this section is divided into two parts, initially focusing on the optimization of the adsorbing diblock copolymer followed by the complexing one.

#### 3.4.1 Adsorbing Diblock Copolymer: PS-*b*-PAA

Regarding the adsorbing PS-*b*-PAA polymeric micelles, a correct balance between the hydrophobic anchoring core and the hydrophilic extending corona is crucial to maximize the film density while simultaneously providing sufficient stability.^12,44^ It is hypothesized that a higher PS/PAA block ratio would lead to stronger anchoring of the layer because of the larger hydrophobic PS core, while a lower block ratio (*i.e.*, longer PAA chains) would enhance the complexation to PDMAEMA-*b*-PEG diblock copolymers in the second step. To investigate the influence of the PS/PAA block ratio and PAA block length of the PS-*b*-PAA micelles on the properties of the adsorbed primer, four different diblock copolymers were compared: PS_81_-*b*-PAA_81_, PS_32_-*b*-PAA_100_, PS_27_-*b*-PAA_287_, and PS_27_-*b*-PAA_436_, with PS/PAA block ratios of 1.00, 0.32, 0.09, and 0.06, respectively.

**Figure 3:**
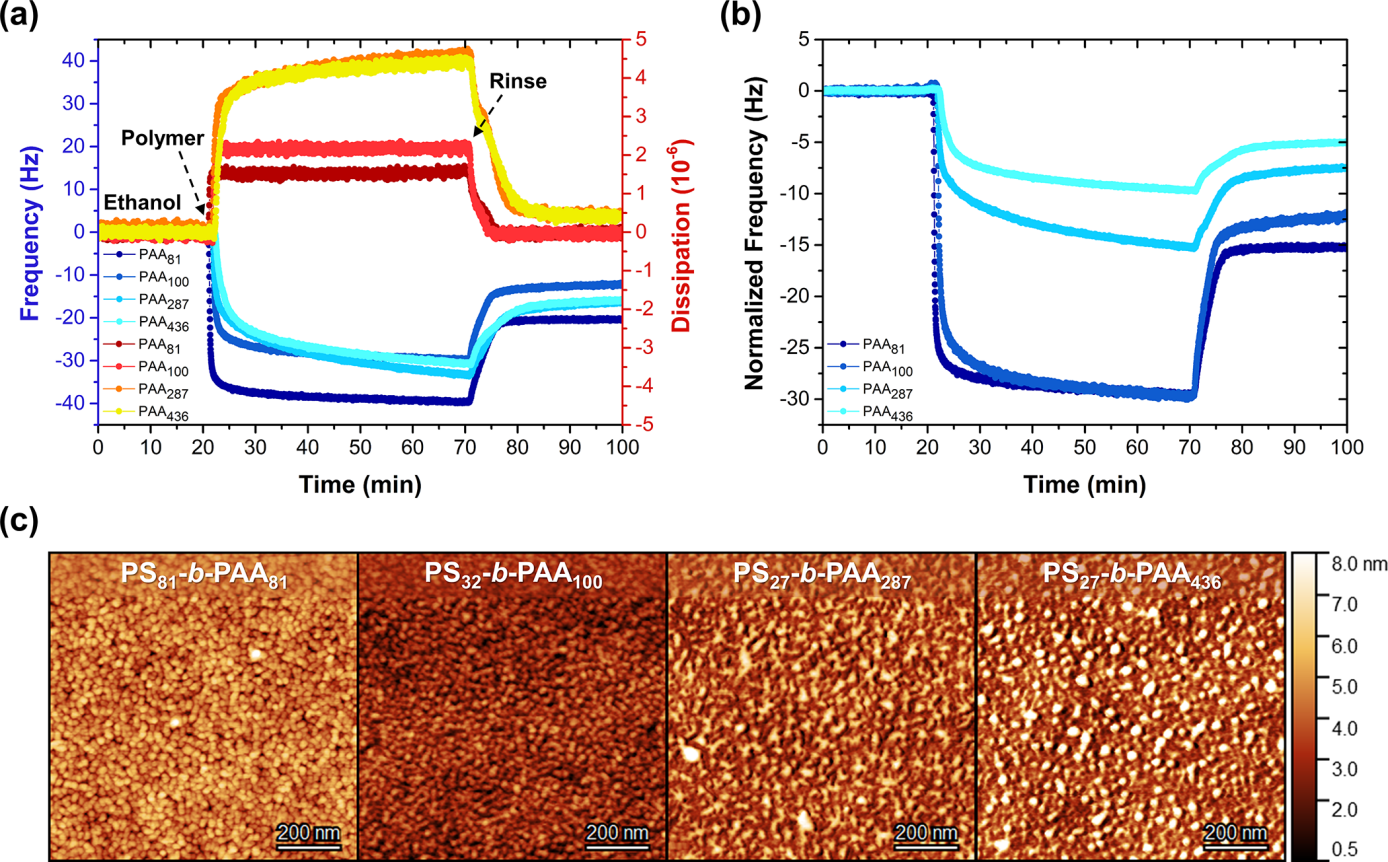
Data displaying the effect of the PS/PAA block ratio and PAA block length on the adsorption behavior and layer characteristics of the formed primers. (a) QCM-D graph showing the *in situ* formation of the PS-*b*-PAA primer layers. (b) Weight-normalized QCM-D graph in which the frequency is normalized by the molecular weight ratio, illustrating the relative number of micelles adsorbing to the surface. (c) Tapping mode AFM height images of the adsorbed PS-*b*-PAA primer layers. The corresponding phase images and cross-sectional profiles are available in the SI (Figure S14).

The adsorption of the self-assembled polymeric micelles was monitored *in situ* by means of a QCM-D (Figure 3a). Irrespective of the PS/PAA block ratio, each copolymer rapidly adsorbs to the PS-coated sensor surface, transforming into a relatively rigid film after rinsing (Δ*D* ≈ 0). An increased PAA chain length (PAA_287_, PAA_436_) endows the coating with a slightly more viscous character, which can be explained by the lengthy and hydrated PAA chains extending outward into the solution. The fitted wet thickness corroborates this finding (Table S6): considering the copolymers with (almost) identical PS block lengths, a longer PAA block leads to an increased wet thickness. Interestingly, at first glance, a change in the block ratio does not invoke a clear trend in the amount of mass adsorbed, as seen from the relatively indistinct frequency shifts after rinsing. However, when compensating for the difference in unit mass for each type of copolymer, the frequency shifts now represent the relative number of polymer units adsorbing to the surface rather than its total mass (Figure 3b). From this graph, the efficiency of binding becomes clear: a higher PS/PAA block ratio (*i.e.*, a smaller PAA corona) facilitates the adsorption of more micelles to the surface. This finding is consistent with the literature: for the core to adsorb, the corona must be compressed and deformed. This energy barrier is easier to overcome when the corona is small.^45,46^

The difference in binding efficiency also manifests itself in the obtained morphologies, as seen in AFM (Figure 3c). The adsorbed PS_81_-*b*-PAA_81_ and PS_32_-*b*-PAA_100_ primer layers with the highest PS/PAA block ratio and smallest PAA corona are characterized by a relatively dense and homogeneous micellar topography, while a low-density, nonuniform film is obtained for adsorbed PS_27_-*b*-PAA_436_. Hence, the surface density increases with an increased block ratio. The dry thickness, grafting density, and surface roughness follow accordingly (Table S6): a higher block ratio increases the dry film thickness and grafting density and minimizes the surface roughness. It is worth mentioning that all primer layers exhibited an increased wettability (68–74°) with respect to the hydrophobic PS substrate (93°), resembling that of a PAA-based film (57–73°).^47−49^ This confirms the anticipated conformation of the adsorbed micelles: the PS cores adsorb to the surface, forcing the PAA chains to stretch outward. Moreover, since an increased PS/PAA block ratio facilitates micelle adsorption, the surface becomes enriched by a greater number of micelles (*i.e.*, a higher concentration of PAA at the interface), resulting in a (slightly) improved wettability of the primer at increased block ratios (Table S6). This will facilitate complexation to the second (antifouling) diblock copolymer.

Overall, it can be concluded that PS-*b*-PAA micelles with a higher PS/PAA block ratio (*i.e.*, a smaller PAA corona) produce the most densely packed, uniformly distributed, and hydrophilic layers, and PS_81_-*b*-PAA_81_ was therefore selected for the successive experiments.

#### 3.4.2 Complexing Diblock Copolymer: PDMAEMA-*b*-PEG

Since the block ratio has been optimized for the adsorbing diblock copolymer (PS_81_-*b*-PAA_81_), the same should be established for the complexing one: PDMAEMA-*b*-PEG. According to data reported previously by de Vos et al. concerning zipper brushes, it is expected that the number of diblock copolymers complexing to the preadsorbed PS_81_-*b*-PAA_81_ primer is determined by full charge compensation between the charges present.^29,30^ As a consequence, it is possible to control the grafting density of the final brush by tuning the PE block ratio between PAA and PDMAEMA: if the PDMAEMA block is (much) smaller in length than the PAA chains (PE block ratio >1), multiple PDMAEMA blocks can bind to a single PAA chain, thereby increasing the grafting density and enhancing its antifouling performance.^11,29,30^ On the contrary, a complexing block that is too small would lack sufficient adsorption energy to produce a stable final coating.

**Figure 4:**
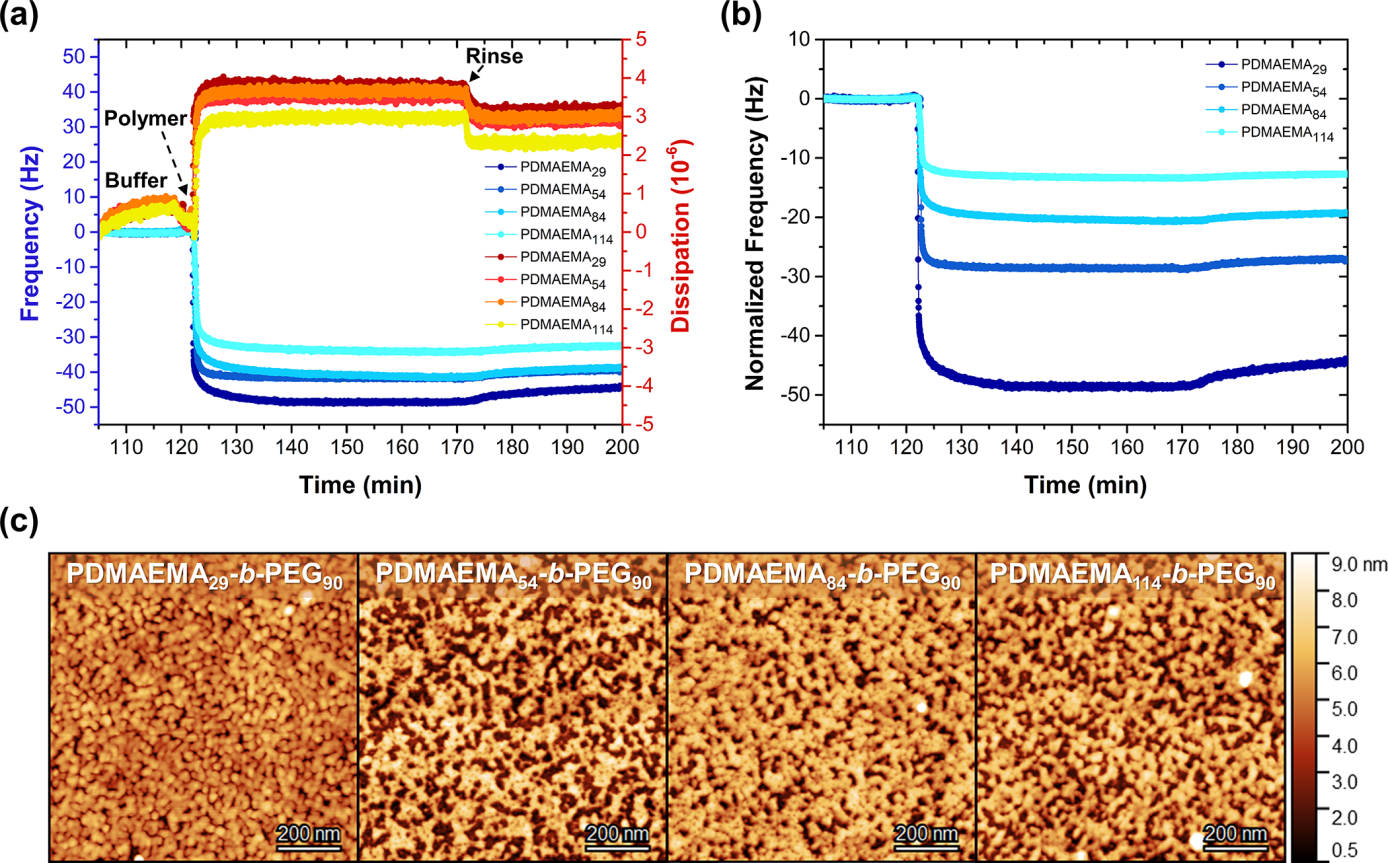
Data displaying the effect of the PE block ratio on the complexation behavior and layer characteristics of the formed zipper brush coatings. (a, b) QCM-D graphs showing the *in situ* complexation of the PDMAEMA_*x*_-*b*-PEG_90_ diblock copolymers to the preadsorbed PS_81_-*b*-PAA_81_ primer (pH 7, 1 mM) (a) before and (b) after normalizing the frequency by molecular weight ratio. (c) Tapping mode AFM height images of the adsorbed PEG-based zipper brush coatings. The corresponding phase images and cross-sectional profiles are available in the SI (Figure S15).

To explore this maximization of grafting density, four PDMAEMA_*x*_-*b*-PEG_90_ diblock copolymers with varying PDMAEMA block lengths (*x* = 29, 53, 84, and 114) were complexed to the PS_81_-*b*-PAA_81_ primer using the optimized buffer conditions (pH 7, 1 mM) (Figure 4a). The corresponding PE block ratios are 2.8, 1.5, 1.0, and 0.7. Once the copolymer reaches the primer, complexation occurs rapidly and stabilizes almost instantaneously, independent of the PE block ratio. The negligible change in frequency during rinsing and the net positive dissipation shift suggest the formation of a strongly bound and hydrated zipper brush. The efficiency of complexation, however, is strongly correlated to the chosen block ratio, which is best illustrated by the weight-normalized graph of Figure 4b: decreasing the PDMAEMA block length increases the efficiency of binding, which would suggest an increase in the grafting density.

Depending on the PDMAEMA block length, the obtained brushes were either characterized by a too low grafting density to be defined as a true “brush”, or were found to be within the mushroom-to-brush transition regime, which occurs for grafting densities higher than 0.05 nm^–2^ (Table S7 and Section 2). The AFM height images clearly depict an increase in surface coverage when complexing to copolymers with smaller PDMAEMA block lengths (*i.e.*, higher PE block ratios) (Figure 4c). Additionally, a higher PE block ratio increases the final film thickness and grafting density, minimizes surface roughness, and improves the wettability (Table S7). The zipper brush containing the smallest PDMAEMA block (*x* = 29) has a noticeably lower contact angle (52.4°) than the other three zipper brushes (63.3–67.5°), which implies that more PEG chains are positioned at the interface, thereby corroborating the aforementioned hypothesis of having a higher grafting density. Still, all zipper brushes exhibited only a slightly increased hydrophilicity with regard to the PS-*b*-PAA primer, rather representing a PDMAEMA film (65°)^50^ than a PEG-based film (36–39°).^51^ In our previous work, it was hypothesized that the root cause for this low wettability could be related to an incorrect conformation of several PDMAEMA-*b*-PEG copolymer chains during complexation, where PEG chains interact with the preadsorbed PAA chains via hydrogen bonding, thereby positioning the positively charged PDMAEMA chains at the interface.^31,52^ On the other hand, none of the brushes managed to attain full charge compensation (Table S7), indicating that free PAA chains (θ = 57–73°) may still dominate the interface, which provides another explanation for the relatively high contact angles seen.

In fact, the calculated charge compensation of the current brushes is unexpectedly low compared to previous work on zipper brushes reported by de Vos et al. in which they showed complete charge compensation.^29,30^ We believe there are two possible explanations for this discrepancy in charge compensation. First of all, desorption processes may occur during the complexation step: if the electrostatic interaction between preadsorbed PS-*b*-PAA and oppositely charged PDMAEMA-*b*-PEG is sufficiently strong, it may initiate their release from the surface into solution. This process is indiscernible in QCM-D since desorption will be a relatively minor process compared to the simultaneously occurring complexation of chains. As a consequence, the number of PS-*b*-PAA chains available for complexation is effectively lower than the initially calculated grafting density of the primer would suggest. This would also explain the relatively small increase in the film thickness after complexation, measured by ellipsometry. In other words, the thickness of the second layer may in reality be appreciably larger than the specified value, which consequently equals a charge compensation higher than currently indicated. Moreover, ellipsometry may fail to accurately determine the dry thickness of the coating: to properly fit the data, the software assumes a homogeneous thin film, which is an incorrect assumption regarding the rough coatings as evidenced by AFM (Figure 4c and Table S7). Due to the unreliability of the employed methods and calculations, it was decided to neglect further assessment of charge compensation for all experiments discussed onward.

**Figure 5:**
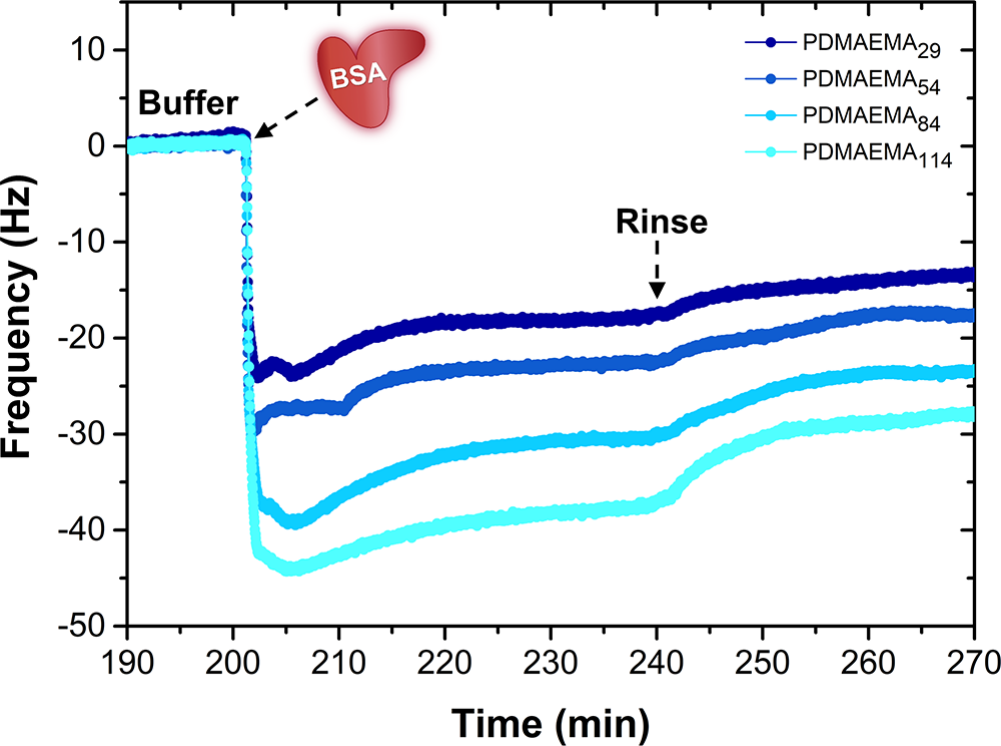
QCM-D graph presenting the antifouling performance of the adsorbed PEG-based zipper brush coatings tested against BSA (pH 7, 1 mM).

Finally, the antifouling performance of each zipper brush was tested against negatively charged BSA (Figure 5). None of the adsorbed zipper brushes were able to fully suppress its adhesion (Δ*f* ≠ 0), which could be ascribed to an insufficient brush density or uniformity and/or the presence of residual charge. However, protein attachment was more effectively reduced for zipper brushes containing smaller PDMAEMA blocks (*i.e.*, having a relatively higher grafting density).

Hence, by tuning the PDMAEMA block length (*i.e.*, the PE block ratio), the number of blocks binding to a single PAA chain can be controlled, which is directly correlated to the acquired grafting density and, therefore, its antifouling performance.

### 3.5 Choice of Antifouling Block

**Figure 6:**
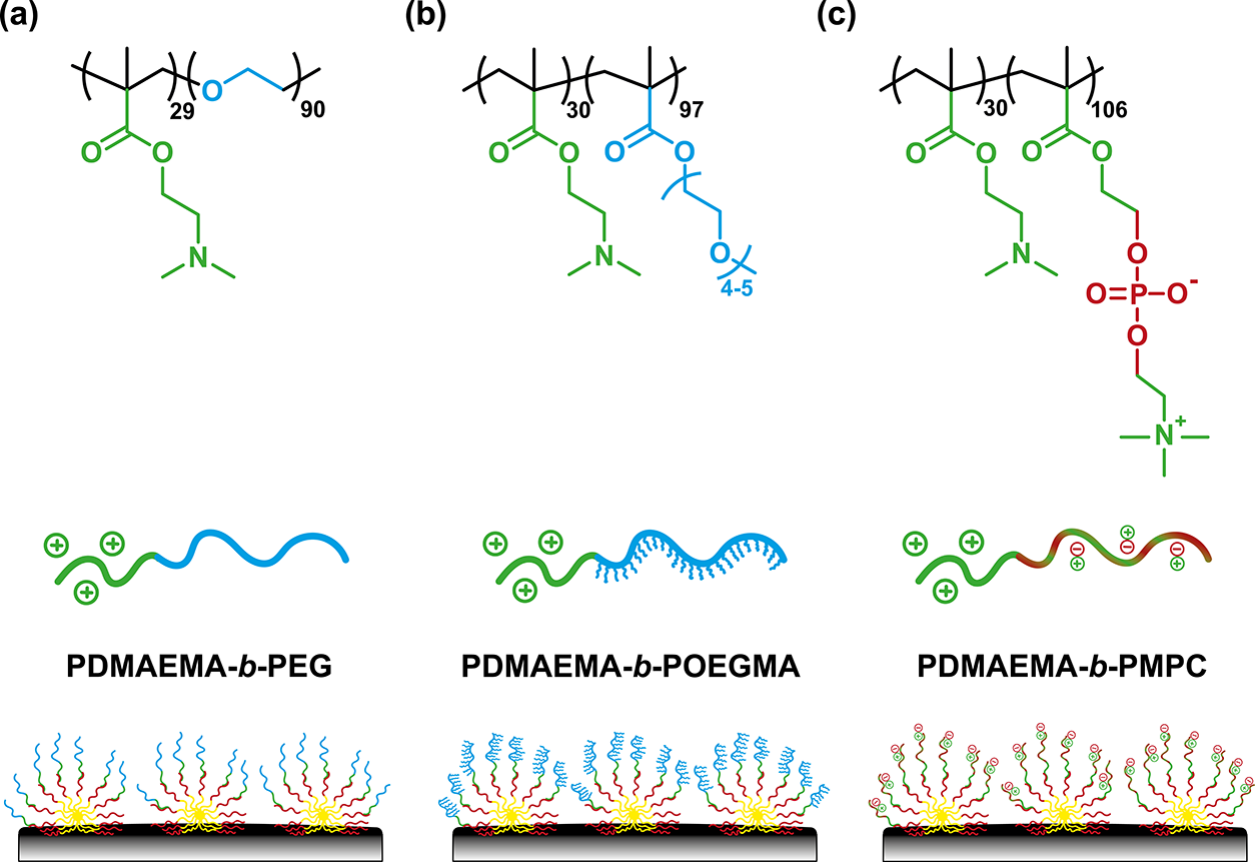
Schematic representation of the utilized antifouling diblock copolymers and the corresponding adsorbed zipper brushes, including (a) PDMAEMA-*b*-PEG, (b) PDMAEMA-*b*-POEGMA, and (c) PDMAEMA-*b*-PMPC. The diblock copolymers have comparable compositions (*i.e.*, block ratio and length) but differ in the excluded volume and ionic nature.

Even though the adsorbed PEG-based zipper brush had been optimized considerably through tuning of the pH, salt concentration, and polymer block ratios, it still resulted in substantial attachment of BSA. The final optimization strategy therefore involved switching to a different antifouling block of comparable composition (*i.e.*, block ratio and length) (Figure 6). While PEG has been considered the golden standard for decades, owing to its charge-neutral and hydrated character,^53,54^ poly(oligo(ethylene glycol) methyl ether methacrylate) (POEGMA) possesses similar properties, but with a bulkier comb-like architecture. This could enhance the internal brush density to generate an even more impenetrable layer.^55−58^ Alternatively, zwitterionic poly(2-methacryloyloxyethyl phosphorylcholine) (PMPC) can strongly bind eight water molecules per monomer unit (instead of one for PEG) and its hydration shell is formed by ion-dipole interactions, which are much stronger than hydrogen bonds, therefore creating a stronger energetic barrier toward approaching foulants.^13,53,59^ Hence, both POEGMA- and PMPC-based brushes may demonstrate an enhanced barrier against fouling relative to that of the PEG-based brush.

**Figure 7:**
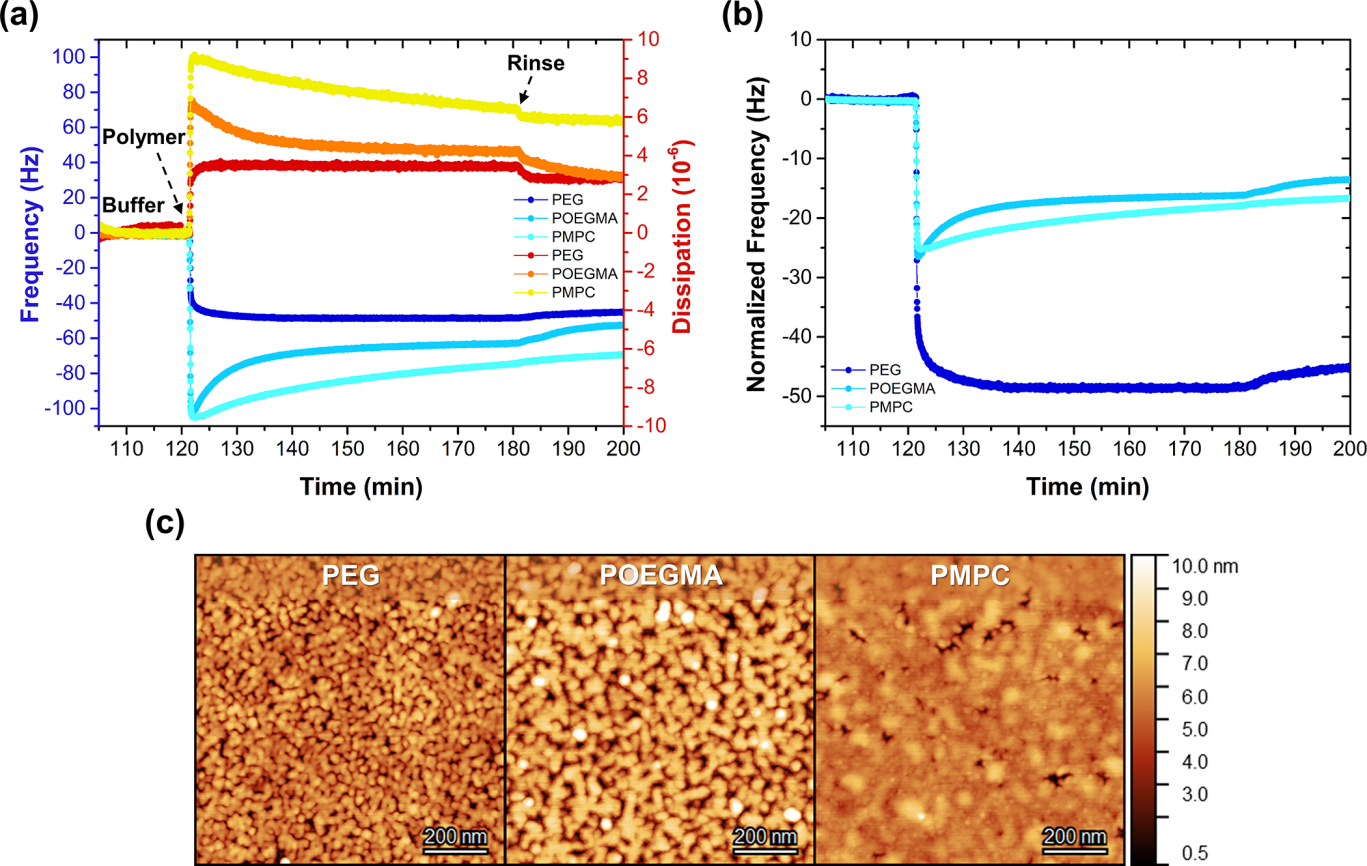
Data displaying the effect of the antifouling block on the complexation behavior and layer characteristics of the formed zipper brush coatings. (a, b) QCM-D graphs showing the *in situ* complexation of the antifouling diblock copolymers to the preadsorbed PS_81_-*b*-PAA_81_ primer (pH 7, 1 mM) (a) before and (b) after normalizing the frequency by molecular weight ratio. (c) Tapping mode AFM height images of the adsorbed zipper brush coatings with different antifouling blocks. The corresponding phase images and cross-sectional profiles are available in the SI (Figure S18).

The complexation of each diblock copolymer to the PS_81_-*b*-PAA_81_ primer was monitored *in situ* by using QCM-D (Figure 7a). In contrast to the quick complexation and stabilization of the PEG-containing polymers, the POEGMA- and PMPC-based polymers required a longer time to complex and equilibrate, as evidenced by the slow increment in frequency over time. For POEGMA, the rate of complexation could be impeded by the increased steric hindrance induced by its bulky comb-like architecture, while for PMPC, it could be related to the thermodynamically unfavorable disruption of the electrostatically induced hydration shell accompanying it. The presence of a tightly bound hydration layer formed by ionic solvation of the charged PMPC groups is confirmed by the significant positive shift in dissipation, indicating the formation of a more viscous and hydrated layer. In accordance with the frequency creeping up, the dissipation slowly decreases over time, which could indicate the loss of bound counterions and water molecules, and/or a reduced chain flexibility due to rearrangements at the surface, resulting in a more collapsed and rigid structure. According to the net frequency shifts, it appears that the efficiency of complexation of the PMPC copolymers surpasses that of the other two. However, the binding efficiency is best illustrated by the weight-normalized graph in Figure 7b, which actually suggests a superior complexation of the PEG-containing polymers. Comparison of the calculated grafting densities indeed confirms a significantly (3×) higher grafting density for the PEG zipper brush (Table S8). The PMPC and POEGMA brushes have a similar, but lower grafting density than PEG, as was expected based on sterics (POEGMA) and extensive hydration (PMPC).

According to the AFM images (Figure 7c), the surface coverage looks slightly higher for the PEG brush with respect to the POEGMA brush and is characterized by a lower surface roughness (1.1 *vs* 1.7 nm). The PMPC brush, however, distinguishes itself from the other two: its surface morphology reveals a remarkably homogeneously covered film. We believe the highly hydrated PMPC copolymers may more easily spread upon complexation, thereby covering a larger surface area. Independent of the choice of antifouling block, all obtained zipper brushes have a highly hydrated character, indicated by the significant wet thickness, and they result in similarly thick coatings after drying (Table S8). However, the surface roughness decreases and the wettability increases when changing the antifouling block from POEGMA, to PEG, to PMPC, which is in accordance with the AFM data. The PEG and POEGMA zipper brushes exhibit a slightly more hydrophilic character (52.4 and 60.9°) than the PS-*b*-PAA primer layers, rather representing an intermediate between a PAA film (57–73°)^47−49^ and a PEG-based (36–39°)^51^ or a POEGMA-based (44°) film, respectively.^60,61^ The PMPC zipper brush is characterized by an exceedingly higher wettability, but the contact angle (31°) is not nearly as low as the one recorded for covalently grafted PMPC brushes (<3°).^62,63^ The relatively high contact angles may be explained by the available PAA chains still remaining at the interface, which is expected based on the low grafting density.

The surface zeta potential (ZP) was recorded at each stage of the zipper brush formation using the streaming potential technique (Figure S19). According to these measurements, all surfaces, including the zipper brushes, were characterized by a net negative surface charge. However, it should be emphasized that the employed technique calculates the absolute values by assuming that the surfaces are uniformly charged and are homogeneously covered, something which is impossible to achieve for the current adsorbed brushes.^64^ Hence, it was decided to ignore the recorded absolute values and focus on the trends instead. As expected, the ZP decreases substantially after the first adsorption step, caused by the negatively charged and polar PAA chains contained within the PS-*b*-PAA primer. The ZP increases after the complexation step, which can be explained by the hydrophilic and (net) charge-neutral chains now dominating the interface. Interestingly, even though the choice of the antifouling block offered control over the grafting density, roughness, and wettability, it did not seem to affect the surface potential: all zipper brushes were characterized by a comparable ZP.

**Figure 8:**
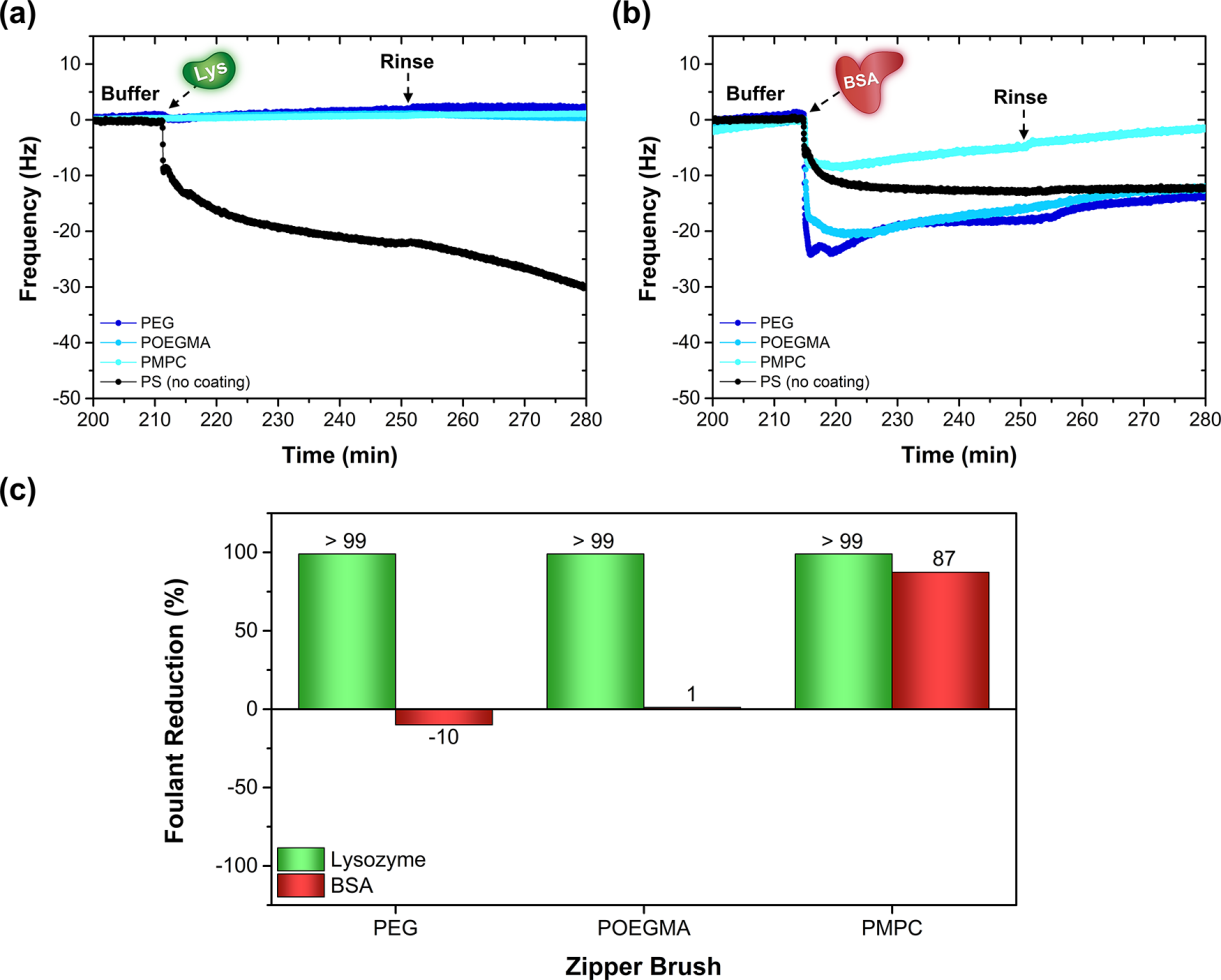
QCM-D graphs summarizing the *in situ* antifouling performance of the adsorbed zipper brush coatings with different antifouling blocks against (a) lysozyme and (b) BSA (pH 7, 1 mM). (c) Bar graph representing the antifouling efficacy of the zipper brushes with respect to the pristine PS-coated substrate.

Finally, the antifouling efficacy of each zipper brush was tested against two fouling agents with varying characteristics and sizes: positively charged lysozyme (14 kDa, pI 9.7) and negatively charged BSA (66 kDa, pI 4.5).^41^ According to the QCM-D data, all zipper brushes successfully suppressed the attachment of lysozyme (Δ*f* ≈ 0), as opposed to the pristine polystyrene substrate (Figure 8a). Even though the zwitterionic PMPC chain bears both positive and negative charges, the equal number of charges makes it electrically neutral and, therefore, it does not attract lysozyme. Interestingly, in the case of BSA, only the PMPC zipper brush was able to convincingly outperform the PS benchmark (Figure 8b). These differences become even more striking when normalizing the frequency shifts with regard to the pristine PS-coated substrate and converting the deviations into a bar graph (Figure 8c). Even though their grafting densities were lower, both the POEGMA and PMPC brushes have an increased potency against BSA adhesion than the PEG brush, presumably due to the enhanced internal density of the POEGMA brush and the electrostatically induced hydration layer of the zwitterionic PMPC brush.^57,59^ The superior antifouling performance of the PMPC brush can additionally be explained by its higher surface coverage and uniformity (evidenced by AFM), as well as its high wettability (confirmed by its low CA and high Δ*D*).

Hence, replacing the antifouling PEG block with zwitterionic PMPC significantly improves the antifouling performance of the resulting adsorbed zipper brush.

## 4 Conclusions and Outlook

The antifouling performance of the two-step adsorbed zipper brush was optimized via systematic tuning of various parameters, including pH, salt concentration, and polymer design. By using a dissolving medium with a neutral pH and a low ionic strength of 1 mM, brushes with improved antifouling properties were obtained. Adsorption of polymeric micelles with a higher PS/PAA block ratio and a smaller PAA corona (*i.e.*, PS_81_-*b*-PAA_81_) resulted in the most densely packed, uniform, and hydrophilic primer layers, as these micelles had to overcome a smaller deformation energy in order to adsorb. By tuning the PE block ratio, the number of diblock copolymers binding to a single PAA chain could be maximized (PAA/PDMAEMA ≈ 3), resulting in zipper brushes with the highest grafting density and wettability, which enabled an increased suppression against BSA adsorption. Finally, changing the antifouling block from linear PEG to comb-like POEGMA or zwitterionic PMPC led to a further enhancement of the antifouling properties, presumably due to the increased internal density of the POEGMA brush and the strong electrostatically induced hydration layer of the PMPC brush. The latter specifically showed a superior antifouling performance (>99% lysozyme, 87% BSA), which can be attributed to its higher surface coverage and uniformity as well as its significantly hydrated character.

Overall, the optimization strategies employed have led to a low-density PMPC-based zipper brush with a considerable antifouling efficacy, which, combined with its straightforward application strategy, could become an attractive contender for future antifouling coatings produced on hydrophobic surfaces. Additionally, the incorporated salt- and pH-sensitive PE complex may endow the adsorbed PMPC brush with a triggered reversibility property, allowing easy regeneration of the (contaminated) brush without the need of tedious cleaning protocols: rinsing with either a low/high pH solution or a high ionic strength solution should facilitate the disintegration and removal of the top complexed layer. Subsequent regeneration of the brush *via* a one-step complexation procedure should permit the preparation of a new, fully functional antifouling coating. Further research is required to investigate the potential triggered reversibility of these two-step adsorbed zipper brushes as well as their stability under varying solvent conditions (*e.g.*, pH, salinity, temperature, and static/dynamic flow) over an extended period of time. Finally, alternative strategies need to be explored to further enhance the grafting density and reach charge neutrality, as these factors ultimately determine the antifouling efficacy of the brush.

## Supporting Information Available

Polymer synthesis and analysis (^1^H NMR, GPC, and ATR-FTIR); theoretical brush analysis, including essential formulas to calculate specific brush characteristics; and additional data regarding the main results (DLS spectra, QCM-D graphs, AFM images, CA images, and a ZP bar graph) (PDF)
