# Supplementary material for: Toward Effective and Adsorption-Based Antifouling Zipper Brushes: Effect of pH, Salt, and Polymer Design
Source: ACS Appl Polym Mater. 2023 Sep 14;5(10):7968–81. doi: 10.1021/acsapm.3c01217 (PMC10580283; doi:10.1021/acsapm.3c01217)
Supplement: Supplementary file 1 — ap3c01217_si_001.pdf [file ap3c01217_si_001.pdf]

Supporting Information

Toward Effective and Adsorption-Based Antifouling Zipper Brushes: Effect of pH, Salt, and Polymer Design

Anna M. C. Maan[a], Anton H. Hofman[a], Théophile Pelras[b], Ilan M. Ruhof[a], Marleen Kamperman[a*], and Wiebe M. de Vos[c*]

[a]Polymer Science, Zernike Institute for Advanced Materials, University of Groningen, Nijenborgh 4, 9747 AG Groningen, The Netherlands.

[b]Macromolecular Chemistry and New Polymeric Materials, Zernike Institute for Advanced Materials, University of Groningen, Nijenborgh 4, 9747 AG Groningen, The Netherlands.

[c]Membrane Science and Technology, MESA+ Institute for Nanotechnology, University of Twente, P.O. Box 217, 7500 AE Enschede, The Netherlands.

*Email: marleen.kamperman@rug.nl
*Email: w.m.devos@utwente.nl

Table of Contents

1. Polymer Synthesis and Analysis 3
1.1. Materials 3
1.2. Characterization 3
1.3. Synthesis and Analysis 4
1.3.1. RAFT Synthesis of PS-*b*-PAA Diblock Copolymers 4
1.3.2. RAFT Synthesis of $PDMAEMA_{30}$-*b*-$POEGMA_{97}$ 10
1.3.3. RAFT Synthesis of $PDMAEMA_{30}$-*b*-$PMPC_{106}$ 14
1.3.4. Synthesis of PEG-*b*-PDMAEMA Diblock Copolymers by ATRP 16

2. Theoretical Brush Analysis 19
2.1. Grafting Density 19
2.2. Reduced Tethered Density 19
2.3. Charge Compensation 20

3. Additional Data 20

References 27

1. Polymer Synthesis and Analysis

1.1. Materials

Azobisisobutyronitrile (AIBN, 98%), aluminum oxide (Al_2O_3, basic, activated), copper(I) bromide (CuBr, 98%), 2-(dodecylthiocarbonothioylthio)-2-methylpropionic acid (DDMAT, 98%), 1,4-dioxane (≥99.0%), 2-(dimethylamino)ethyl methacrylate (DMAEMA, hydroquinone stabilized, 98%), 1,1,4,7,10,10-hexamethyltriethylenetetramine (HMTETA, 97.0%), *N,N*-dimethylformamide (DMF, ≥99.9%), deuterated chloroform ($CDCl_3$, 99.8% D), deuterated dimethyl sulfoxide (DMSO-d_6, 99.5% D), oligo(ethylene glycol) methyl ether methacrylate (OEGMA, M_n = 300 g mol^{-1}), 2-methacryloyloxyethyl phosphorylcholine (MPC, 97%), and methanol-d_4 (≥99.8%) were purchased from Sigma-Aldrich. Styrene (4-*tert*-butylcatechol stabilized, 99.0%) was purchased from Acros Organics. Methanol (≥99.9%), *n*-pentane (99%), diethyl ether (Et_2O, HPLC grade), isopropanol (≥99.8%), *n*-hexane (99%), and acetone (HPLC grade) were purchased from Macron Fine Chemicals. Anisole (≥99%) was sourced from Merck. Tetrahydrofuran (THF, BHT stabilized, ≥99.8%) and 1,1,1,3,3,3-hexafluoro-2-propanol (HFIP, ≥99.8%) were purchased from Biosolve. Poly(ethylene glycol) monomethylether (PEG_{90}, M_n = 4.01 kg mol^{-1}, $Đ$ = 1.05) and *tert*-butyl acrylate (*t*BA, MEHQ stabilized, 98%) were purchased from TCI. Hydrochloric acid solution (HCl, 37%) was sourced from Boom. The dialysis tubing (Spectra/Por 6, MWCO = 1 kDa) was purchased from Spectrum Chemicals.

The 2-cyanopropan-2-yl propyl trithiocarbonate (CPP-TTC) chain transfer agent (CTA) and PEG_{90}-Br macroinitiator were synthesized as reported elsewhere.[1,2]

AIBN was recrystallized twice from methanol. All commercially available monomers were passed over a short basic aluminum oxide column to remove the inhibitor directly before their use in the polymerizations, except for MPC. All other chemicals were used as received.

1.2. Characterization

Proton Nuclear Magnetic Resonance (^{1}H NMR) Spectroscopy. ^{1}H NMR spectra were recorded on an Agilent 400-MR 400 MHz spectrometer operating at room temperature. Polymer samples were dissolved in the appropriate deuterated solvent ($CDCl_3$, DMSO-d_6, or methanol-d_4) at concentrations of approximately 20 mg mL^{-1}. The samples were measured using a pulse width of 45 µs, a spectral width of 12/-2 ppm or 14/-2 ppm (for PAA-containing polymers), a recycle delay of 1 s, and taking 32 scans. The obtained spectra were analyzed using MestreNova software (version 14.2).

Gel Permeation Chromatography (GPC). GPC was performed on a Viscotek GPCMax system equipped with model 302 TDA detectors and two columns (PolarGel L and M, 8 µm 30 cm). The columns and detectors were maintained at a constant temperature of 50 °C. DMF containing 0.01 M LiBr salt was used as the eluent at a flow rate of 1 mL min^{-1}. Narrow poly(methyl methacrylate) (PMMA) standards from Polymer Standards Service were used to calibrate the system. All polymer samples were dissolved in the corresponding eluent at

concentrations of approximately 2–3 mg mL^{-1} and were filtered over a 0.20 μm PTFE filter prior to injection. Data acquisition and calculations were performed according to conventional calibration using Viscotek Omnisec software (version 5.0).

Attenuated Total Reflection-Fourier Transform Infrared (ATR-FTIR) Spectroscopy. ATR-FTIR spectra were recorded on a Bruker Vertex 70 spectrometer equipped with an ATR diamond single reflection module. Spectra were acquired in the range of 4000–400 cm^{-1} with a spectral resolution of 2 cm^{-1} and 64 scans per run. The collected data was analyzed using OPUS software (version 7.5), in which atmospheric compensation and baseline corrections were applied to the recorded spectra.

1.3. Synthesis and Analysis

1.3.1. RAFT Synthesis of PS-*b*-PAA Diblock Copolymers

1. Synthesis of the PS Macro-CTAs

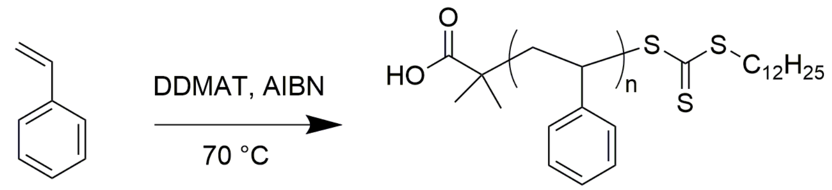

Scheme S1. Reaction scheme for the synthesis of PS macro-CTAs.

Polystyrene macro-CTAs were synthesized according to an adapted literature procedure.[3] The reaction conditions for obtaining PS macro-CTAs with distinct lengths are summarized in **Table S1**. Purified styrene, DDMAT and AIBN were charged in a 25 mL round-bottom flask equipped with a stirring bar and a septum. Once all chemicals were dissolved, the yellow reaction mixture was sparged with nitrogen for 10 minutes. The flask was immersed in a thermostated oil bath at 70 °C while stirring continuously. After the indicated reaction time, the reaction mixture was quenched by cooling the flask in cold water and subsequently exposing it to air. A ^1H NMR sample was prepared in order to calculate the conversion through comparison of the monomer and polymer peaks. The viscous yellow polymer mixture was purified by precipitating twice from methanol: the polymer mixture was first diluted with a small amount of THF (~2 mL) and precipitated dropwise into a beaker containing 500 mL of thoroughly stirred methanol. The precipitated product was collected by vacuum filtration, washed with methanol, and air-dried on the filter for at least an hour. The polymer product was redissolved in THF and the precipitation procedure was repeated. The precipitated polymer was collected and dried in a vacuum oven (40 °C) overnight. The yield of the yellow powder was determined and the product was characterized by ^1H NMR (**Figure S1a**), GPC (**Figure S1b**), and ATR-FTIR (**Figure S4b**).

^1H NMR (400 MHz, $CDCl_3$): δ (ppm) = 7.30-6.30 (br, 5 CH, aromatic ring), 3.26 (br, S-CH_2, CTA), 2.30-1.70 (br, CH), 1.70-1.30 (br, CH_2), 1.27 (br, $C_{10}H_{20}$, CTA), 0.89 (br, CH_3, CTA).

Table S1. Reaction conditions for the synthesis of PS macro-CTAs by RAFT polymerization. The subscripts denote the degree of polymerization. Amounts are given in mmol, reaction times (t_R) are in hours, conversions were determined by ^{1}H NMR (%), yields are given in %, and molecular weights (M_n) are reported in kg mol^{-1}. $M_{n,calc}$ represents the calculated molecular weight based on the initial concentrations and monomer conversion. $M_{n,GPC}$ and the molecular weight distribution (*Đ*) were determined by GPC.

Polymer	Styrene	DDMAT	AIBN	t_R	Conv.	Yield	$M_{n,calc}$	$M_{n,GPC}$	*Đ*
PS_{27}	261<br>(96 eq.)	2.7<br>(1 eq.)	0.36<br>(0.13 eq.)	7	28.2	90.8	3.2	3.2	1.14
PS_{32}	39.9<br>(36 eq.)	1.1<br>(1 eq.)	0.12<br>(0.11 eq.)	18	87.9	76.2	3.7	3.5	1.10
PS_{81}	36.7<br>(135 eq.)	0.27<br>(1 eq.)	0.031<br>(0.11 eq.)	23	60.3	52.0	8.8	8.1	1.09
PS_{85}	36.7<br>(136 eq.)	0.27<br>(1 eq.)	0.031<br>(0.11 eq.)	22	63.3	49.4	9.2	8.3	1.09

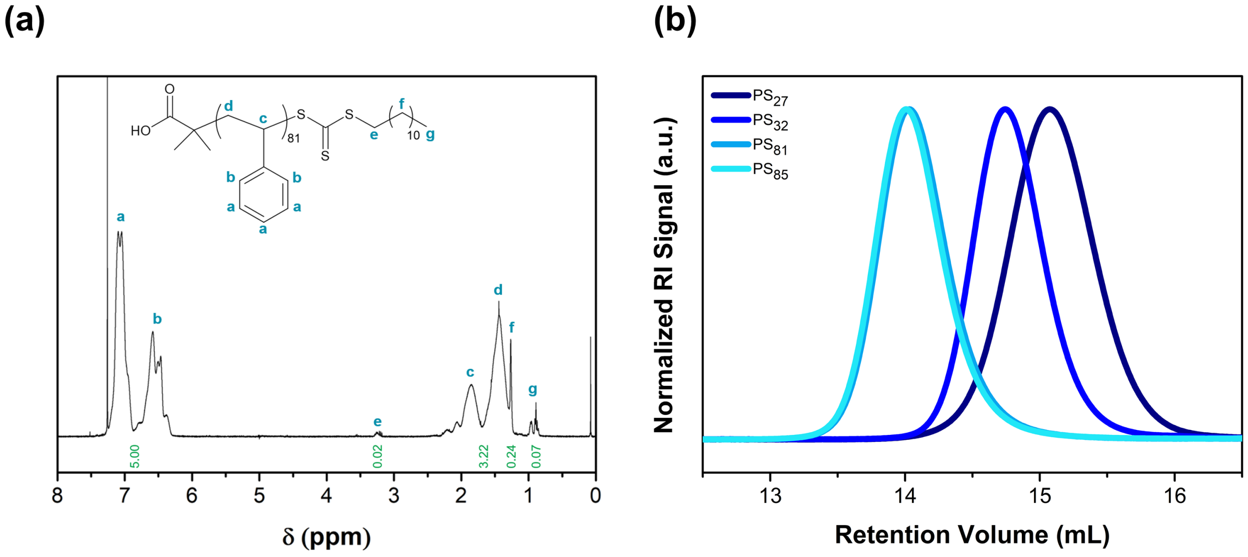

Figure S1. (a) ^{1}H NMR spectrum ($CDCl_3$) of a typical PS macro-CTA (PS_{81}). (b) GPC chromatograms (DMF) of all synthesized PS macro-CTAs: PS_{27}, PS_{32}, PS_{81}, and PS_{85}.

2. Synthesis of PS-*b*-P*t*BA

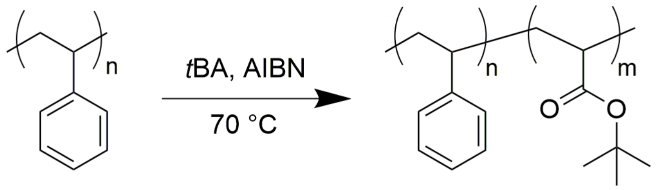

Scheme S2. Reaction scheme for the synthesis of PS-*b*-P*t*BA diblock copolymers.

The reaction conditions for obtaining PS-*b*-P*t*BA diblock copolymers with distinct block lengths and ratios are summarized in **Table S2**. PS macro-CTA, purified *tert*-butyl acrylate (*t*BA), AIBN (using a 1.0 mg mL^{-1} stock solution in 1,4-dioxane/anisole/THF), and 1,4-dioxane/anisole/THF were charged in a 20 mL glass vial and mixed until everything was dissolved. After complete dissolution, the yellow mixture was carefully transferred to a 25 mL round-bottom flask equipped with a stirring bar and septum. The reaction mixture was sparged with nitrogen for 10 minutes. The flask was immersed in a thermostated oil bath at 70 °C. After the indicated reaction time, the reaction mixture was quenched by cooling the flask in cold water and subsequently exposing it to air. A ^{1}H NMR sample was prepared in order to calculate the conversion through comparison of the polystyrene and *t*BA peaks. The viscous yellow polymer mixture was purified by precipitating the undiluted solution into 500 mL of thoroughly stirred methanol. The precipitated product was collected by vacuum filtration, washed with methanol, air-dried on the filter, and further dried in a vacuum oven (40 °C) overnight. The yield of the slightly yellowish powder was determined and the product was characterized by ^{1}H NMR (**Figure S2a**), GPC (**Figure S2b** and **Figure S3**), and ATR-FTIR (**Figure S4b**).

^{1}H NMR (400 MHz, $CDCl_3$): δ (ppm) = 7.30-6.30 (br, 5 CH, PS aromatic ring), 3.33 (br, S-CH_2, CTA), 2.35-1.10 (br, CH and CH_2, backbone PS and P*t*BA), 1.44 (s, -$C(CH_3)_3$, P*t*BA), 1.26 (br, $C_{10}H_{20}$, CTA), 0.90 (br, CH_3, CTA).

Table S2. Reaction conditions for the synthesis of PS-*b*-P*t*BA diblock copolymers by RAFT polymerization. The subscripts denote the degree of polymerization of each block. Amounts of reactants are given in mmol and solvents in mL. Reaction times (t_R) are in hours, conversions were determined by ^{1}H NMR (%), yields are given in %, and molecular weights (M_n) are reported in kg mol^{-1}. f_{PtBA} represents the weight fraction of P*t*BA and $M_{n,calc}$ is the sum of the calculated molecular weights based on the initial concentrations and monomer conversion ($M_{n,PS}$ + $M_{n,PtBA}$). $M_{n,GPC}$ and the molecular weight distribution (*Đ*) were determined by GPC.

Polymer	PS-CTA	*t*BA	AIBN	Solvent	t_R	Conv.	Yield	f_{PtBA}	$M_{n,calc}$	$M_{n,GPC}$	*Đ*
PS_{32}-*b*-P*t*BA_{100}	0.14 (1 eq.)	14.4 (104 eq.)	0.012 (0.086 eq.)	5.0 (dioxane)	21	95.9	72.9	0.79	16.5	12.4	1.27
PS_{27}-*b*-P*t*BA_{287}	0.047 (1 eq.)	23.5 (499 eq.)	0.0049 (0.11 eq.)	10.3 (anisole)	6	57.7	8.2	0.93	40.0	29.8	1.20
PS_{27}-*b*-P*t*BA_{338}	0.063 (1 eq.)	27.7 (440 eq.)	0.0061 (0.10 eq.)	10.0 (dioxane)	5.5	76.4	55.6	0.94	46.5	39.0	1.30
PS_{27}-*b*-P*t*BA_{436}	0.047 (1 eq.)	23.4 (499 eq.)	0.0049 (0.11 eq.)	7.7 (THF)	23	87.2	14.5	0.95	59.1	31.5	1.79
PS_{81}-*b*-P*t*BA_{79}	0.038 (1 eq.)	3.10 (81 eq.)	0.0042 (0.11 eq.)	1.1 (dioxane)	23	96.2	62.3	0.55	18.9	15.1	1.17
PS_{81}-*b*-P*t*BA_{81}	0.10 (1 eq.)	8.55 (82 eq.)	0.012 (0.12 eq.)	2.9 (dioxane)	22	98.8	56.6	0.55	19.2	15.6	1.15
PS_{85}-*b*-P*t*BA_{81}	0.12 (1 eq.)	9.75 (82 eq.)	0.013 (0.11 eq.)	3.3 (dioxane)	23	99.4	79.3	0.54	19.6	15.8	1.15

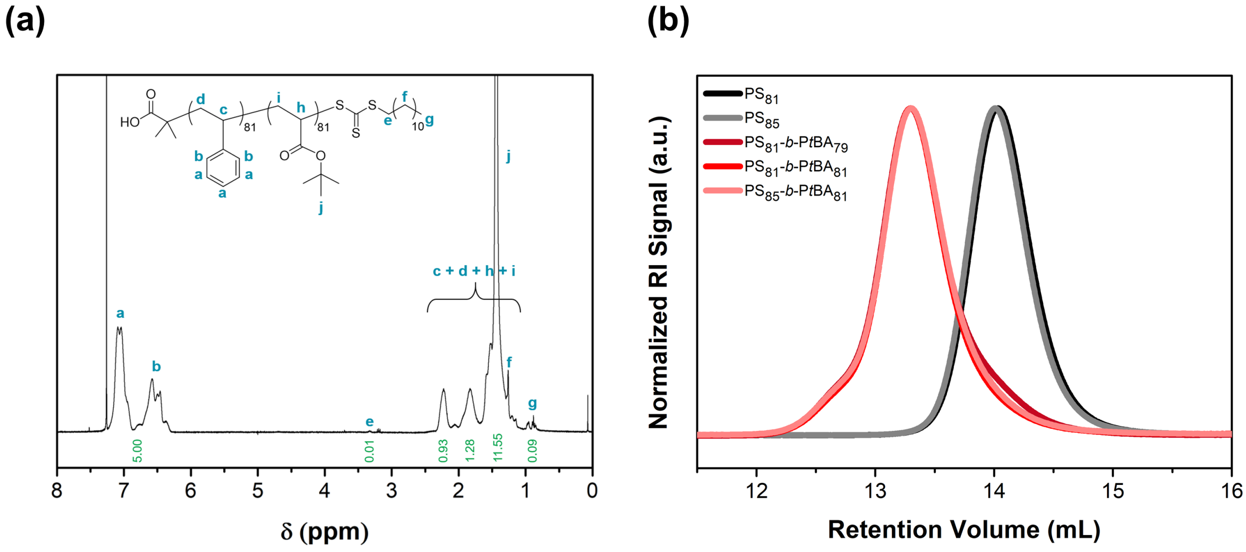

Figure S2. (a) ^{1}H NMR spectrum ($CDCl_3$) of a typical PS-*b*-P*t*BA diblock copolymer (PS_{81}-*b*-P*t*BA_{81}). (b) GPC chromatograms (DMF) of the $PS_{81/85}$ macro-CTAs (black) and the $PS_{81/85}$-*b*-P*t*$BA_{79/81}$ diblock copolymers (red).

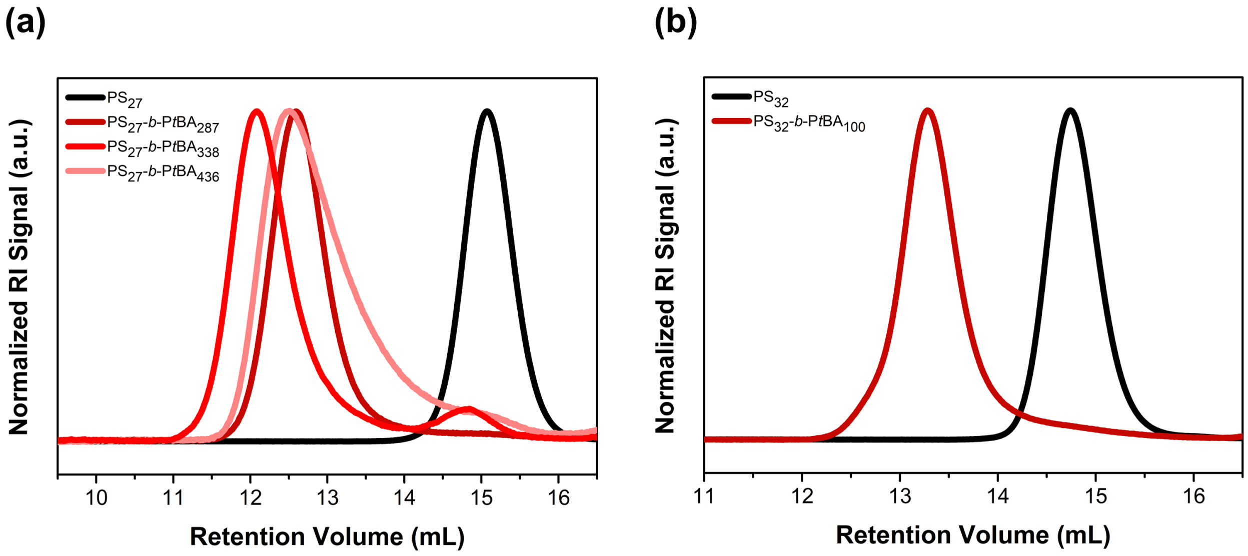

Figure S3. GPC chromatograms (DMF) of (a) the PS_{27} macro-CTA (black) and the PS_{27}-*b*-P*t*BA diblock copolymers (red) and (b) the PS_{32} macro-CTA (black) and the PS_{32}-*b*-P*t*BA_{100} diblock copolymer (red).

3. Deprotection of PS-*b*-P*t*BA

HFIP/HCl

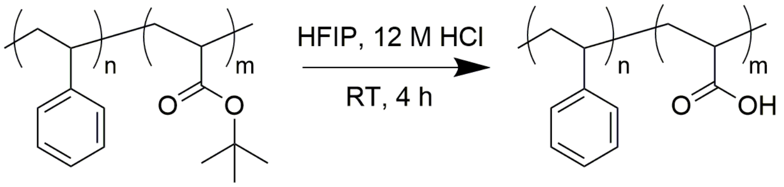

Scheme S3. Reaction scheme for the deprotection of PS-*b*-P*t*BA diblock copolymers containing a short PS block.

The deprotection of PS-*b*-P*t*BA diblock copolymers containing a short PS block (PS_{27} and PS_{32}) was performed according to a previously reported procedure.[4]

PS-*b*-P*t*BA (1 g, 1 eq. *t*BA) was charged in a 250 mL round-bottom flask and dissolved in 100 to 130 mL of 1,1,1,3,3,3-hexafluoro-2-propanol (HFIP). Once dissolved, 12 M HCl (1.3 eq.) was added dropwise to the stirred polymer solution. After 4 hours, the solvent was removed *in vacuo* using a rotary evaporator and the obtained product was redissolved in THF (8–10 mL) and precipitated twice into 500 mL of stirred *n*-pentane. The precipitated product was collected by vacuum filtration and dried in a vacuum oven (40 °C) overnight. The yield of the slightly yellowish powder was determined (88-93%) and the product was characterized by ^1H NMR (**Figure S4a**) and ATR-FTIR (**Figure S4b**).

^1H NMR (400 MHz, DMSO-d_6): δ (ppm) = 12.22 (s, COOH), 7.40-6.10 (br, 5 CH, PS aromatic ring), 2.40-1.00 (br, CH and CH_2, backbone PS and PAA).

Dioxane/HCl + reflux

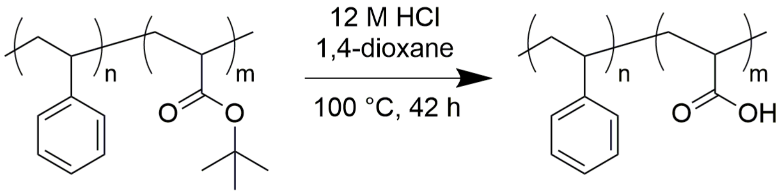

Scheme S4. Reaction scheme for the deprotection of PS-*b*-P*t*BA diblock copolymers containing a long PS block.

Due to the insolubility of long PS blocks in HFIP, a different deprotection method was followed for the $PS_{81/85}$-containing diblock copolymers.[5,6]

PS-*b*-P*t*BA (1 g, 1 eq. *t*BA) was dissolved in 12 mL of 1,4-dioxane in a 50 mL round-bottom flask equipped with a stirring bar and reflux condenser. After dissolution, 12 M HCl (5 eq.) was added to the stirred solution and the mixture was heated to 100 °C. After 42 hours, the mixture was cooled down, the solvent was removed *in vacuo* using a rotary evaporator, and a ^{1}H NMR spectrum was recorded to confirm the successful deprotection. The deprotected polymer was redissolved in 10 mL of 1,4-dioxane and precipitated into 500 mL of stirred *n*-pentane. The precipitated brownish powder was collected by vacuum filtration and dried in a vacuum oven (40 °C) overnight. The yield of the light brown powder was determined (88%) and the product was characterized by ^{1}H NMR (**Figure S4a**) and ATR-FTIR (**Figure S4b**).

<u>^{1}H NMR (400 MHz, DMSO-*d*$_6$):</u> *δ* (ppm) = 12.22 (s, COOH), 7.40-6.10 (br, 5 CH, PS aromatic ring), 2.40-1.00 (br, CH and CH_2, backbone PS and PAA).

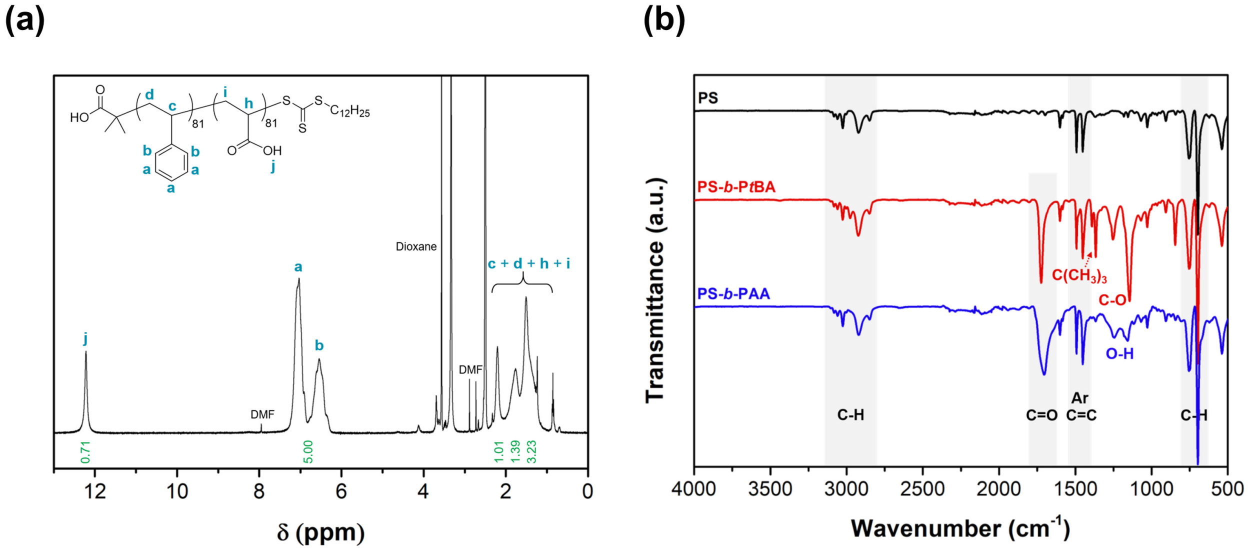

Figure S4. (a) ^{1}H NMR spectrum (DMSO-*d*$_6$) of a typical PS-*b*-PAA diblock copolymer (PS_{81}-*b*-PAA_{81}). (b) ATR-FTIR spectra of the synthesized PS_{81} macro-CTA (black) and the PS_{81}-*b*-P*t*BA_{81} (red) and PS_{81}-*b*-PAA_{81} (blue) diblock copolymers.

1.3.2. RAFT Synthesis of PDMAEMA$_{30}$-*b*-POEGMA$_{97}$

1. Synthesis of the PDMAEMA$_{30}$ Macro-CTA

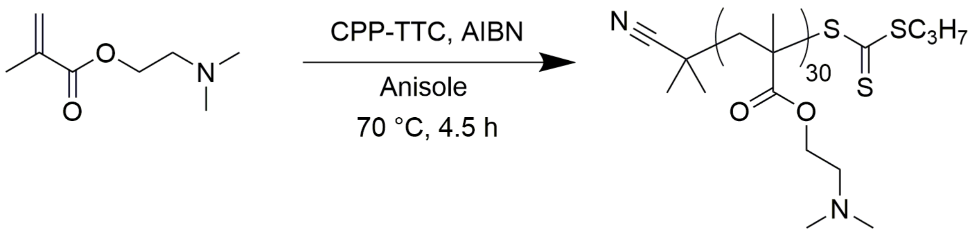

Scheme S5. Reaction scheme for the synthesis of the PDMAEMA$_{30}$ macro-CTA.

Purified 2-(dimethylamino)ethyl methacrylate (DMAEMA) (50 eq., 22.9 mmol, 3.59 g), CPP-TTC (1 eq., 0.461 mmol, 101 mg), AIBN (0.1 eq., 0.047 mmol, 7.7 mg), and anisole (9.9 mL) were charged in a 25 mL round-bottom flask equipped with a stirring bar and septum. After complete dissolution, the reaction mixture was sparged with argon for 15 minutes. A t = 0 h ^{1}H NMR sample was taken toward the end of the degassing cycle. The flask was immersed in a thermostated oil bath at 70 °C. After 4.5 hours, the reaction mixture was quenched by cooling the flask in cold water and subsequently exposing it to air. A t = 4.5 h ^{1}H NMR sample was prepared in order to calculate the conversion through comparison of the anisole standard and DMAEMA peaks (conv. = 61%). The reaction mixture was purified by precipitation into cold *n*-hexane, after which it was collected by vacuum filtration. The polymer product was redissolved in 1,4-dioxane and the precipitation procedure was repeated twice. The polymer was dried at room temperature under high vacuum overnight. The yield of the fine yellow powder was determined (1.67 g, 74.2%) and the product was characterized by ^{1}H NMR (**Figure S5**), GPC (**Figure S6b**), and ATR-FTIR (**Figure S7**).

^{1}H NMR (400 MHz, $CDCl_3$): δ (ppm) = 4.06 (br, O-CH_2), 3.23 (t, S-CH_2, CTA), 2.56 (br, N-CH_2), 2.28 (s, 2x N-CH_3), 2.1-1.7 (br, CH_2, backbone), 1.2-0.7 (br, CH_3, backbone). Conversion = 61%, $M_{n,NMR}$ = 4.9 kg mol^{-1}, $P_{n,NMR}$ = 30.

GPC (DMF): $M_{n,GPC}$ = 7.2 kg mol^{-1}, $Đ$ = 1.17.

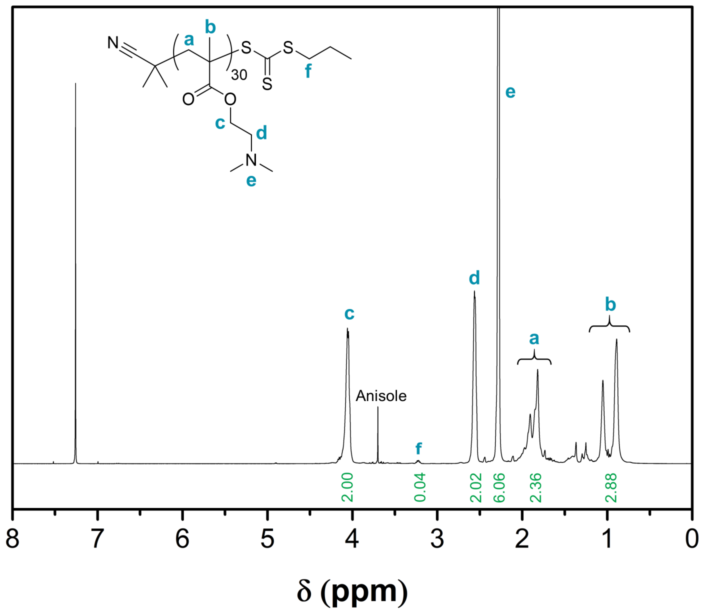

Figure S5. ^{1}H NMR spectrum ($CDCl_3$) of the $PDMAEMA_{30}$ macro-CTA.

2. Synthesis of PDMAEMA$_{30}$-*b*-POEGMA$_{97}$

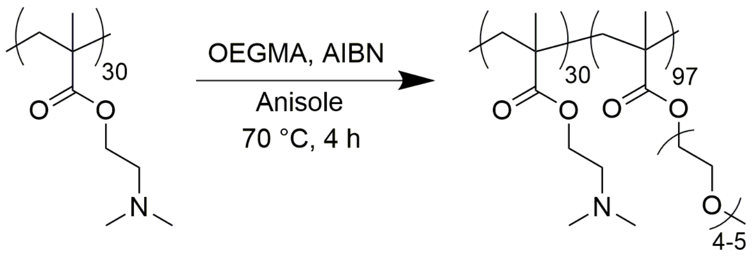

Scheme S6. Reaction scheme for the synthesis of the PDMAEMA$_{30}$-*b*-POEGMA$_{97}$ diblock copolymer.

PDMAEMA$_{30}$ macro-CTA (1 eq., 0.0407 mmol, 199 mg), oligo(ethylene glycol) methyl ether methacrylate (OEGMA) (166 eq., 6.75 mmol, 2.03 g), AIBN (0.1 eq., 0.0042 mmol, 0.70 mg – using 92 mg of a 7.6 mg mL^{-1} stock solution in anisole), and anisole (800 eq., 5.76 mL) were charged in a 25 mL round-bottom flask equipped with a stirring bar and septum. After complete dissolution, the reaction mixture was sparged with argon for 10 minutes. A t = 0 h ^{1}H NMR sample was taken toward the end of the degassing cycle. The flask was immersed in a thermostated oil bath at 70 °C. After 4 hours, the reaction mixture was quenched by cooling the flask in cold water and subsequently exposing it to air. A t = 4 h ^{1}H NMR sample was prepared in order to calculate the conversion through comparison of the anisole standard and OEGMA peaks (conv. = 55%). The reaction mixture was purified by precipitation into cold *n*-hexane, after which it was collected by vacuum filtration. The polymer product was redissolved in acetone and the precipitation procedure was repeated twice. The polymer was dried at room temperature under high vacuum overnight. The yield of the yellow soft solid was determined (1.24 g, 99%) and the product was characterized by ^{1}H NMR (**Figure S6a**), GPC (**Figure S6b**), and ATR-FTIR (**Figure S7**).

^{1}H NMR (400 MHz, $CDCl_3$): δ (ppm) = 4.08 (br, COO-CH_2), 3.65 (br, CH_2, POEGMA), 3.55 (br, CH_2, POEGMA), 3.38 (s, O-CH_3, POEGMA), 2.56 (br, CH_2-N PDMAEMA), 2.28 (s, N-$(CH_3)_2$, PDMAEMA), 2.1-1.5 (br, CH_2, backbone), 1.2-0.5 (br, CH_3, backbone). Conversion = 55%, $M_{n,POEGMA}$ = 29.1 kg mol^{-1}, $P_{n,POEGMA}$ = 97. $M_{n,total}$ = 34.0 kg mol^{-1}. x_{POEGMA} = 0.764, f_{POEGMA} = 0.861.

GPC (DMF): $M_{n,GPC}$ = 23.0 kg mol^{-1}, $Đ$ = 1.28.

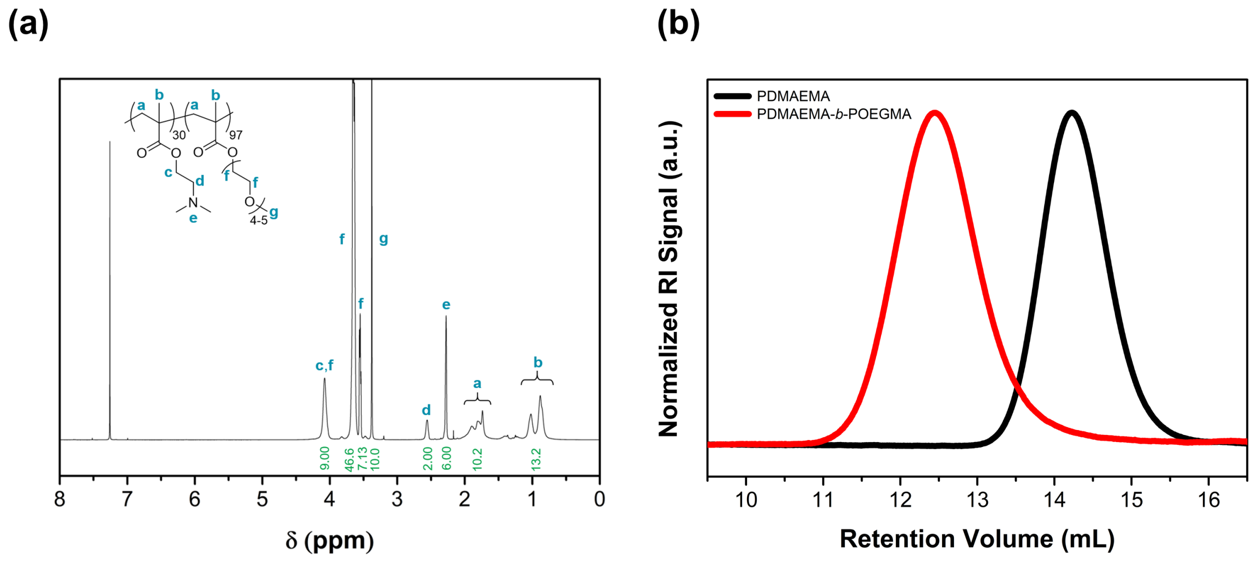

Figure S6. (a) ^{1}H NMR spectrum ($CDCl_3$) of the $PDMAEMA_{30}$-*b*-$POEGMA_{97}$ diblock copolymer. (b) GPC chromatograms (DMF) of the $PDMAEMA_{30}$ macro-CTA (black) and the $PDMAEMA_{30}$-*b*-$POEGMA_{97}$ diblock copolymer (red).

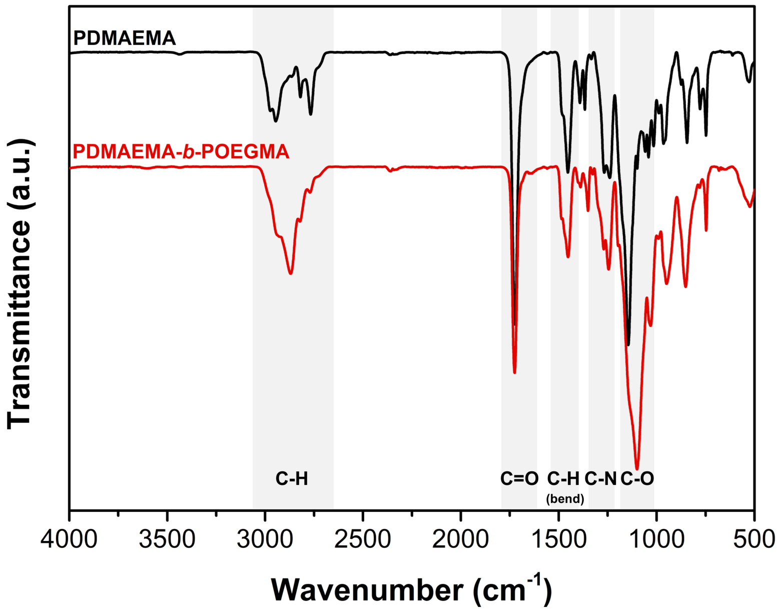

Figure S7. ATR-FTIR spectra of the synthesized $PDMAEMA_{30}$ macro-CTA (black) and the $PDMAEMA_{30}$-*b*-$POEGMA_{97}$ diblock copolymer (red).

1.3.3. RAFT Synthesis of $PDMAEMA_{30}$-*b*-$PMPC_{106}$

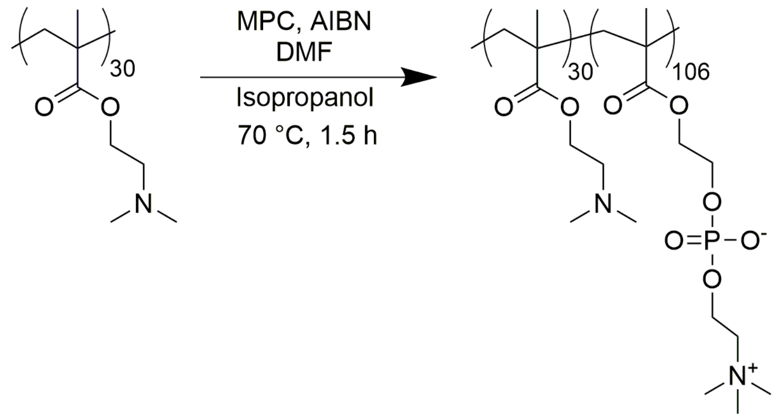

Scheme S7. Reaction scheme for the synthesis of the $PDMAEMA_{30}$-*b*-$PMPC_{106}$ diblock copolymer.

$PDMAEMA_{30}$ macro-CTA (Section 1.3.2) (1 eq., 0.0408 mmol, 200 mg), 2-methacryloyloxyethyl phosphorylcholine (MPC) monomer (151 eq., 6.17 mmol, 1.82 g), AIBN (0.1 eq., 0.0045 mmol, 0.738 mg – using 83.6 mg of an 8.3 mg mL^{-1} stock solution in DMF), DMF (300 eq., 1.34 mL), and isopropanol (900 eq., 4.22 mL) were charged in a 25 mL round-bottom flask equipped with a stirring bar and septum. The solvent mixture of DMF and isopropanol was required to ensure complete dissolution of both PDMAEMA and MPC. The reaction mixture was sparged with argon for 10 minutes. A t = 0 h ^{1}H NMR sample was taken toward the end of the degassing cycle. The flask was immersed in a thermostated oil bath at 70 °C. After 1.5 hours, the reaction mixture was quenched by cooling the flask in cold water and subsequently exposing it to air. A t = 1.5 h ^{1}H NMR sample was prepared in order to calculate the conversion through comparison of the DMF standard and MPC peaks (conv. = 61%). The reaction mixture was purified by precipitation into cold diethyl ether, after which it was redissolved in methanol and dialyzed against methanol/acetone over the course of several days to remove the remainder of unreacted monomer. The polymer solution was concentrated *in vacuo* using a rotary evaporator and dried at room temperature under high vacuum overnight. The yield of the yellow solid was determined (1.21 g, 98.2%) and the product was characterized by ^{1}H NMR (**Figure S8a**) and ATR-FTIR (**Figure S8b**). Due to the unavailability of an aqueous GPC, and undesired interactions between the polymer and the GPC column when dissolved in DMF, characterization *via* GPC was not performed.

<u>^{1}H NMR (400 MHz, methanol-d_4):</u> δ (ppm) = 4.30 (br, O-CH_2, PMPC), 4.19 (br, CH_2-O, PMPC), 4.05 (br, 2x O-CH_2, PDMAEMA and PMPC), 3.71 (br, CH_2-N, PMPC), 3.27 (br, N-$(CH_3)_3$, PMPC), 2.61 (br, CH_2-N, PDMAEMA), 2.30 (s, N-$(CH_3)_2$, PDMAEMA), 2.2-1.6 (br, CH_2, backbone), 1.2-0.5 (br, CH_3, backbone). Conversion = 61%, $M_{n,PMPC}$ = 31.3 kg mol^{-1}, $P_{n,PMPC}$ = 106. $M_{n,total}$ = 36.2 kg mol^{-1}. x_{PMPC} = 0.779, f_{PMPC} = 0.869.

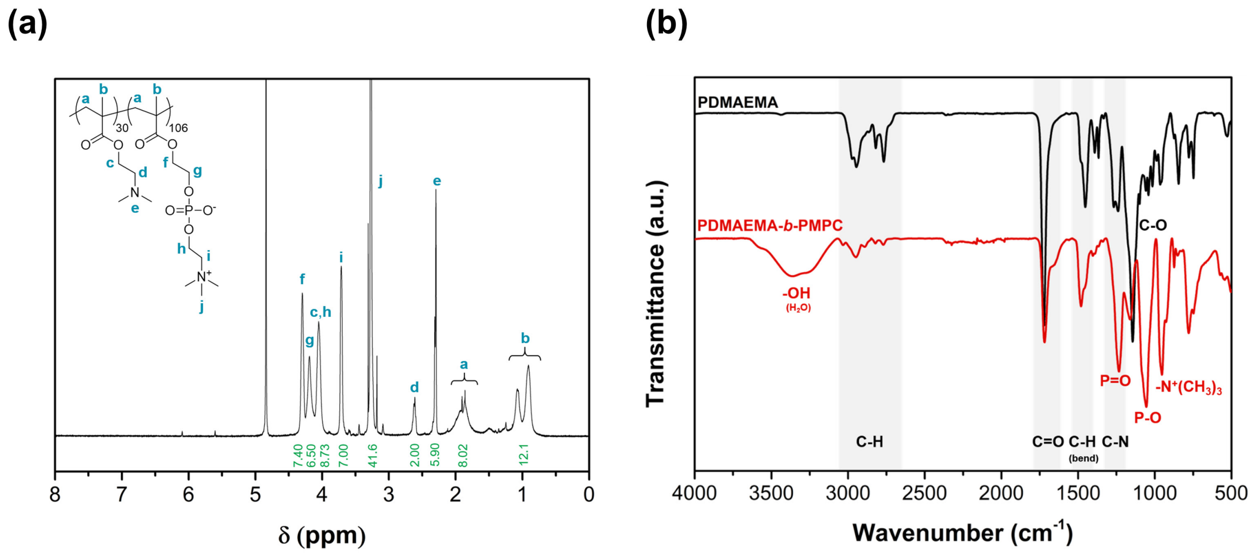

Figure S8. (a) ^{1}H NMR spectrum (methanol-d_4) of the PDMAEMA$_{30}$-*b*-PMPC$_{106}$ diblock copolymer. (b) ATR-FTIR spectra of the synthesized PDMAEMA$_{30}$ macro-CTA (black) and the PDMAEMA$_{30}$-*b*-PMPC$_{106}$ diblock copolymer (red).

1.3.4. Synthesis of PEG-*b*-PDMAEMA Diblock Copolymers by ATRP

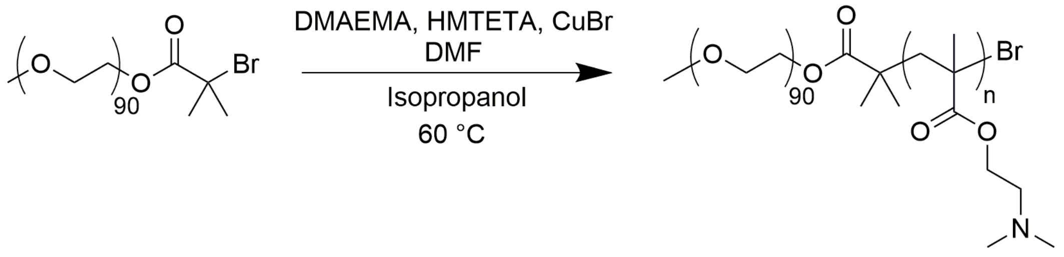

Scheme S8. Reaction scheme for the synthesis of PEG-*b*-PDMAEMA diblock copolymers.

The reaction conditions for obtaining PEG-*b*-PDMAEMA diblock copolymers with distinct block lengths and ratios are summarized in **Table S3**. An overview of all synthesized diblock copolymers and their characteristics can be found in **Table S4**.

PEG_{90} homopolymer (M_n = 4.01 kg mol^{-1}, $Đ$ = 1.05) was purchased from TCI and subsequently converted into a PEG_{90}-Br macroinitiator (M_n = 4.14 kg mol^{-1}, $Đ$ = 1.06) according to a previously reported procedure.[2] PEG_{90}-Br (1 eq.) and isopropanol were charged in a glass vial and carefully heated inside a warm water bath to ensure full dissolution of the PEG_{90}-Br macroinitiator. Purified DMAEMA, 1,1,4,7,10,10-hexamethyltriethylenetetramine (HMTETA, 1 eq.) and DMF (internal standard) were subsequently added to the mixture and everything was mixed until dissolved. The transparent reaction mixture was transferred to a Schlenk flask equipped with a stirring bar and septum, and a t = 0 h ^{1}H NMR sample was taken. The reaction mixture was deoxygenated *via* three freeze-pump-thaw cycles, after which the reaction mixture was frozen once more in order to carefully add the copper(I) bromide (CuBr) catalyst powder (1 eq.) on top, with the argon/vacuum inlet closed. Afterward, the flask was closed with a septum and the freeze-pump-thaw cycle was continued. During the final thaw step, the septum was punctured with a short needle to flush the flask with argon in order to remove any remaining oxygen. Once fully defrosted, the degassing cycle was stopped (removal of outlet needle, closing of inlet valve) and the flask was immersed in a thermostated oil bath at 60 °C to initiate the reaction. After 30 to 60 minutes, the green reaction mixture was quenched by cooling the flask in cold water and subsequently exposing it to air. A ^{1}H NMR sample was prepared in order to calculate the conversion through comparison of the DMF standard and DMAEMA peaks. The reaction mixture was precipitated once in *n*-hexane, redissolved in THF and passed through a short aluminum oxide (basic) column to remove excess CuBr catalyst. The obtained polymer solution was dialyzed against methanol over the course of several days (≥3 days) to remove the remainder of the unreacted monomer and catalyst, while frequently renewing the solvent. The polymer solution was concentrated *in vacuo* using a rotary evaporator and further dried in a vacuum oven (40 °C) overnight. After drying, the yield of the white powder was determined and the product was characterized by ^{1}H NMR (**Figure S9a**), GPC (**Figure S9b**), and ATR-FTIR (**Figure S10**).

<u>^{1}H NMR (400 MHz, $CDCl_3$):</u> δ (ppm) = 4.05 (br, O-CH_2, PDMAEMA), 3.63 (s, 2x CH_2, PEG), 2.55 (br, CH_2-N, PDMAEMA), 2.27 (s, 2x N-CH_3, PDMAEMA), 2.05-1.70 (br, CH_2 backbone, PDMAEMA), 1.16-0.75 (br, CH_3 backbone, PDMAEMA).

Table S3. Reaction conditions for the synthesis of PEG-*b*-PDMAEMA diblock copolymers by ATRP. The subscripts denote the degree of polymerization of each block. Amounts of reactants are given in mmol and solvents in mL. The reaction times (t_R) are in minutes.

Polymer	PEG_{90}-Br	DMAEMA	HMTETA	CuBr	DMF	Isopropanol	t_R
PEG_{90}-*b*-$PDMAEMA_{29}$	0.24<br>(1 eq.)	12.1<br>(50 eq.)	0.24<br>(1 eq.)	0.24<br>(1 eq.)	0.94	7.8	30
PEG_{90}-*b*-$PDMAEMA_{54}$	0.12<br>(1 eq.)	8.45<br>(70 eq.)	0.12<br>(1 eq.)	0.12<br>(1 eq.)	0.65	2.0	60
PEG_{90}-*b*-$PDMAEMA_{84}$	0.12<br>(1 eq.)	24.2<br>(200 eq.)	0.12<br>(1 eq.)	0.12<br>(1 eq.)	1.9	14.8	60
PEG_{90}-*b*-$PDMAEMA_{114}$	0.12<br>(1 eq.)	24.2<br>(200 eq.)	0.12<br>(1 eq.)	0.12<br>(1 eq.)	1.9	22.2	60

Table S4. Overview of the synthesized PEG-*b*-PDMAEMA diblock copolymers. The subscripts denote the degree of polymerization of each block. Conversions were determined by ^{1}H NMR (%), yields are given in %, and molecular weights (M_n) are reported in kg mol^{-1}. $f_{PDMAEMA}$ represents the weight fraction of PDMAEMA and $M_{n,calc}$ is the sum of the calculated molecular weights based on the initial concentrations and monomer conversion ($M_{n,PEG}$ + $M_{n,PDMAEMA}$). $M_{n,GPC}$ and the molecular weight distribution (*Đ*) were determined by GPC.

Polymer	Conv.	Yield	$f_{PDMAEMA}$	$M_{n,calc}$	$M_{n,GPC}$	*Đ*
PEG_{90}-*b*-$PDMAEMA_{29}$	59.1	82.2	0.53	8.7	13.8	1.22
PEG_{90}-*b*-$PDMAEMA_{54}$	75.3	60.2	0.68	12.5	17.2	1.25
PEG_{90}-*b*-$PDMAEMA_{84}$	42.0	ND	0.77	17.3	20.6	1.27
PEG_{90}-*b*-$PDMAEMA_{114}$	38.3	ND	0.82	22.1	23.6	1.25

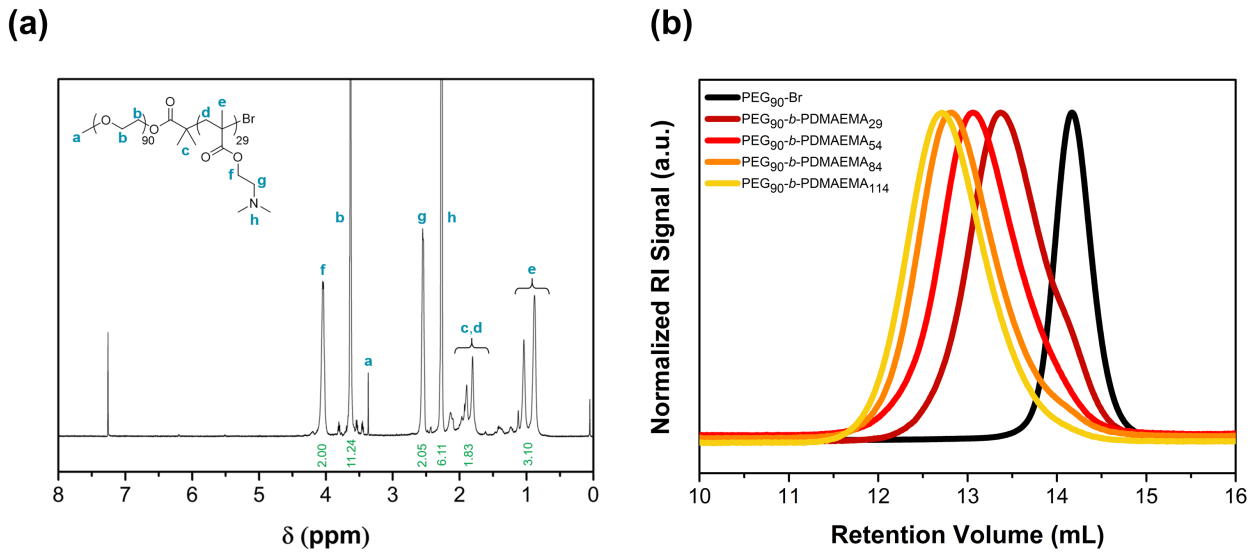

Figure S9. (a) ^{1}H NMR spectrum ($CDCl_3$) of a typical PEG-*b*-PDMAEMA diblock copolymer (PEG_{90}-*b*-$PDMAEMA_{29}$). (b) GPC chromatograms (DMF) of the PEG_{90}-Br macroinitiator (black) and all PEG-*b*-PDMAEMA diblock copolymers (red-yellow).

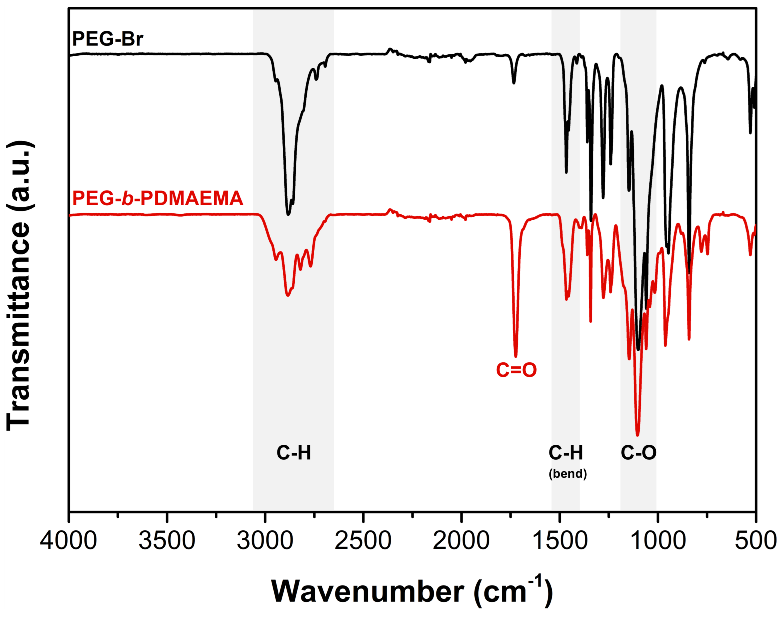

Figure S10. ATR-FTIR spectra of the PEG_{90}-Br macroinitiator (black) and PEG_{90}-*b*-$PDMAEMA_{29}$ diblock copolymer (red).

2. Theoretical Brush Analysis

2.1. Grafting Density

The grafting density (σ) of the adsorbed primer layers and zipper brushes cannot be controlled beforehand such as for surface-initiated brushes, but it can be (roughly) estimated afterward using the following equation:

$$\sigma = \frac{h\ \rho\ N_{AV}}{M_n} \tag{1}$$

where h represents the dry thickness measured by ellipsometry, ρ is the average density, N_{AV} is Avogadro's number (6.022 x 10^{23} mol^{-1}), and M_n is the number-average molecular weight of the polymer chain.[7,8] The molecular weights were determined by ^1H NMR and the average densities of the diblock copolymers were calculated by taking the weight average of the bulk densities of the polymer blocks (**Table 1**). The bulk densities were found to be ρ_{PS} = 1.05 g cm^{-3},[9,10] ρ_{PAA} = 1.41 g cm^{-3},[11] $\rho_{PDMAEMA}$ = 1.32 g cm^{-3},[12,13] ρ_{PEG} = 1.13 g cm^{-3},[14] ρ_{POEGMA} = 1.15 g cm^{-3},[15] and ρ_{PMPC} = 1.30 g cm^{-3}.[16,17] The grafting densities of the primer layers were calculated using the recorded dry thickness, and by assuming that all polymer chains within the adsorbed micelles are exposed. The grafting densities of the zipper brushes were calculated using the difference in dry thickness before and after complexation ($h_{brush} = h_{total} - h_{primer}$), and by assuming there is no intercalation occurring between the individual polymer layers. Due to the many assumptions involved within the grafting density calculations, it should be noted that the presented values can only be considered rough estimates, and may exceed their true values.

2.2. Reduced Tethered Density

End-attached polymer chains can adopt several conformations, including "pancake", "mushroom", "brush", and "highly stretched brush". Depending on the amount of overlap between chains, determined by the area occupied by a single polymer chain ($\propto R_g^2$) and the number of chains per unit area (σ), their conformation can be predicted. This parameter is also known as the reduced tethered density (Σ):

$$\Sigma = \sigma\ \pi\ R_g^2 \tag{2}$$

where σ is the grafting density and R_g is radius of gyration of a tethered chain. According to Brittain and Minko, the transition from the low grafting density "mushroom" to the "brush" regime starts when $\Sigma \approx 1$, and the true brush regime is reached when $\Sigma > 5$.[18]

Assuming a Gaussian polymer coil, the radius of gyration (R_g) for the employed PEG_{90} chain (M_n = 4.4 kg mol^{-1}) equals 2.48 nm.[19] From this, it follows that the polymers start transforming into a brush once reaching grafting densities of 0.05 nm^{-2} or higher, and the true brush regime is reached once $\sigma > 0.25$ nm^{-2}.

2.3. Charge Compensation

Charge compensation was calculated according to the following equation, assuming complete dissociation of all chargeable monomers upon complexation:

$$\text{Charge comp.} = \frac{\sigma_{\text{brush}} \times N_{\text{PDMAEMA}}}{\sigma_{\text{primer}} \times N_{\text{PAA}}} \quad (3)$$

where σ_{primer} and σ_{brush} represent the calculated grafting density of the primer and zipper brush, and N_{PAA} and N_{PDMAEMA} are the lengths of the PAA and PDMAEMA blocks (*i.e.*, the number of monomers).[20] Full charge compensation is reached when the total amount of positive charge equals the total amount of negative charge (*i.e.*, charge comp. = 1).

3. Additional Data

When dispersed in ethanol, the PS-*b*-PAA diblock copolymers self-assemble into micelles with a hydrophobic PS core and a hydrophilic PAA corona. According to the DLS data presented in Figure S11, there is no clear relationship between the polymer composition and the micelle characteristics (*i.e.*, dimensions and polydispersity). This could be explained by the kinetic trapping of the micelles, which arrests the micelle structure evolution.[21] In this case, the dissolution process would affect the size rather than the block ratio and length. On the other hand, it is difficult to accurately predict the relative size of the self-assembled micelles, as it does not only depend on the polymer composition and molecular weight, but on the aggregation number as well.

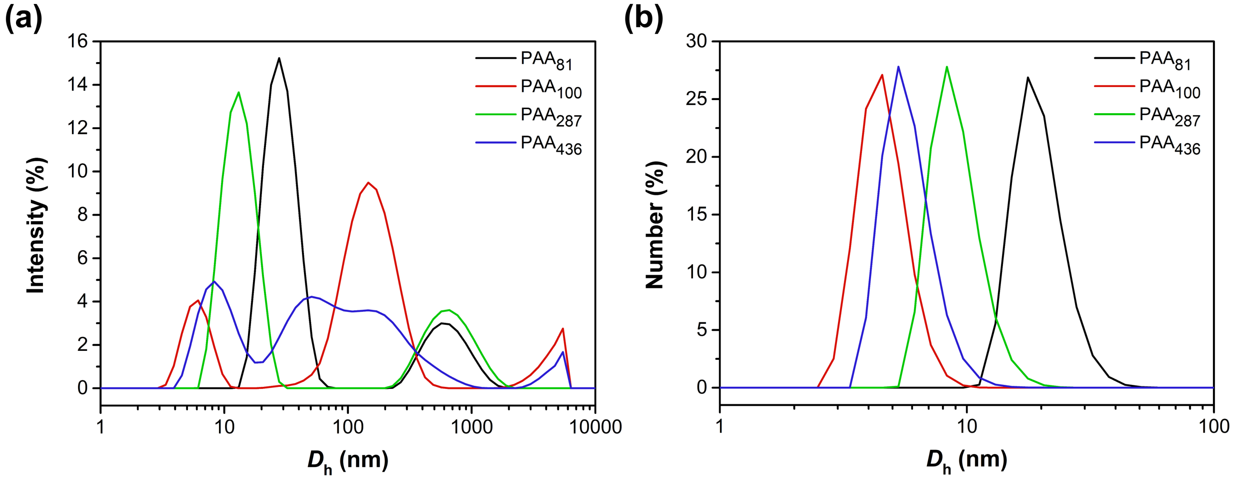

Figure S11. Size distribution plots of the PS-*b*-PAA micelles in ethanol with various block ratios and lengths, including the (a) intensity plot and (b) number plot.

Table S5. Hydrodynamic diameters (D_h) and polydispersity indices (PDI) of the PS-*b*-PAA micelles with various block ratios and lengths.

**Diblock Copolymer**	$\mathbf{D_h}$ (nm) *Intensity*	$\mathbf{D_h}$ (nm) *Volume*	$\mathbf{D_h}$ (nm) *Number*	**PDI** (nm)
PS_{81}-*b*-PAA_{81}	34.0	23.5	19.9	0.34
PS_{32}-*b*-PAA_{100}	112.6	24.3	4.6	0.52
PS_{27}-*b*-PAA_{287}	17.5	10.8	9.2	0.45
PS_{27}-*b*-PAA_{436}	46.2	7.4	5.9	0.53

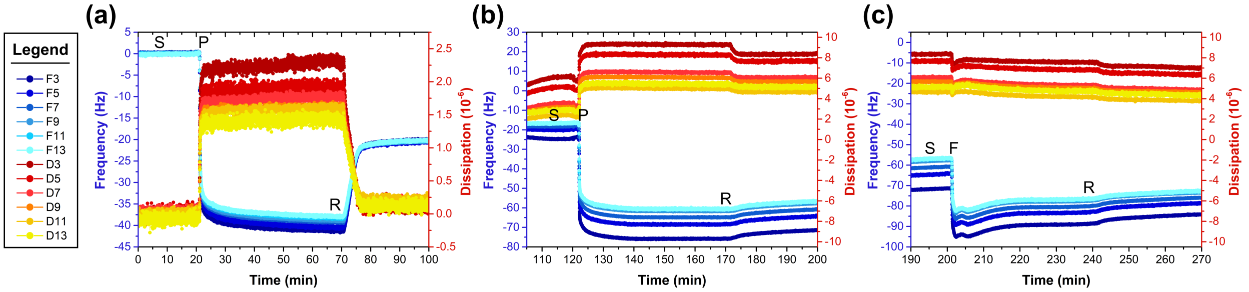

Figure S12. QCM-D graphs showing the *in situ* formation and antifouling performance of a two-step adsorbed PEG-based zipper brush, including (a) the adsorption of PS_{81}-*b*-PAA_{81} micelles, (b) complexation of $PDMAEMA_{29}$-*b*-PEG_{90}, and (c) antifouling testing against BSA. Each step involves equilibration in solvent (S), introduction of polymer (P) or foulant (F), and rinsing (R). The graphs include all harmonic overtones (R3-R13), depicting the total shifts in both frequency (F3-F13, blue) and energy dissipation (D3-D13, red). Due to insufficient energy trapping, the Δf and ΔD values from the fundamental frequency (F1, D1) were usually noisy and were therefore omitted.

Table S6. Summarized characteristics of the adsorbed PS-*b*-PAA films, including the PS/PAA block ratio, the wet thickness calculated from QCM-D (Voigt model), the dry thickness measured by ellipsometry, the calculated grafting density (σ_{primer}), the RMS surface roughness (S_q) obtained from tapping mode AFM, and the recorded static contact angles (θ) in water. The grafting densities were calculated according to the method described in Section 2 of the SI. The captured contact angle images are shown in Figure S13 below. Each denoted value represents the mean ± standard deviation calculated based on at least two (ellipsometry), three (CA), or six (AFM) different spots on the coated sensors, while taking into account the precision and accuracy of each analysis method.

**Diblock Copolymer**	**Block Ratio**	**Wet Thickness** (nm)	**Dry Thickness** (nm)	$\boldsymbol{\sigma_{primer}}$ (nm^{-2})	$\mathbf{S_q}$ (nm)	$\boldsymbol{\theta}$ (°)
PS_{81}-*b*-PAA_{81}	1.00	4.6	2.5 (± 0.1)	0.12	0.9 (± 0.1)	68.3 (± 0.7)
PS_{32}-*b*-PAA_{100}	0.32	2.2	1.1 (± 0.1)	0.08	0.7 (± 0.1)	71.2 (± 3.7)
PS_{27}-*b*-PAA_{287}	0.09	4.5	0.9 (± 0.2)	0.03	1.2 (± 0.1)	75.4 (± 2.9)
PS_{27}-*b*-PAA_{436}	0.06	9.3	0.8 (± 0.1)	0.02	1.5 (± 0.1)	74.0 (± 2.1)

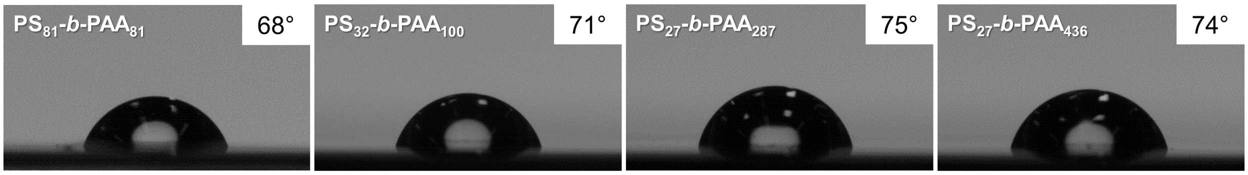

Figure S13. Static contact angles of the adsorbed PS-*b*-PAA films with various block ratios and lengths, representing the intrinsic water wettability.

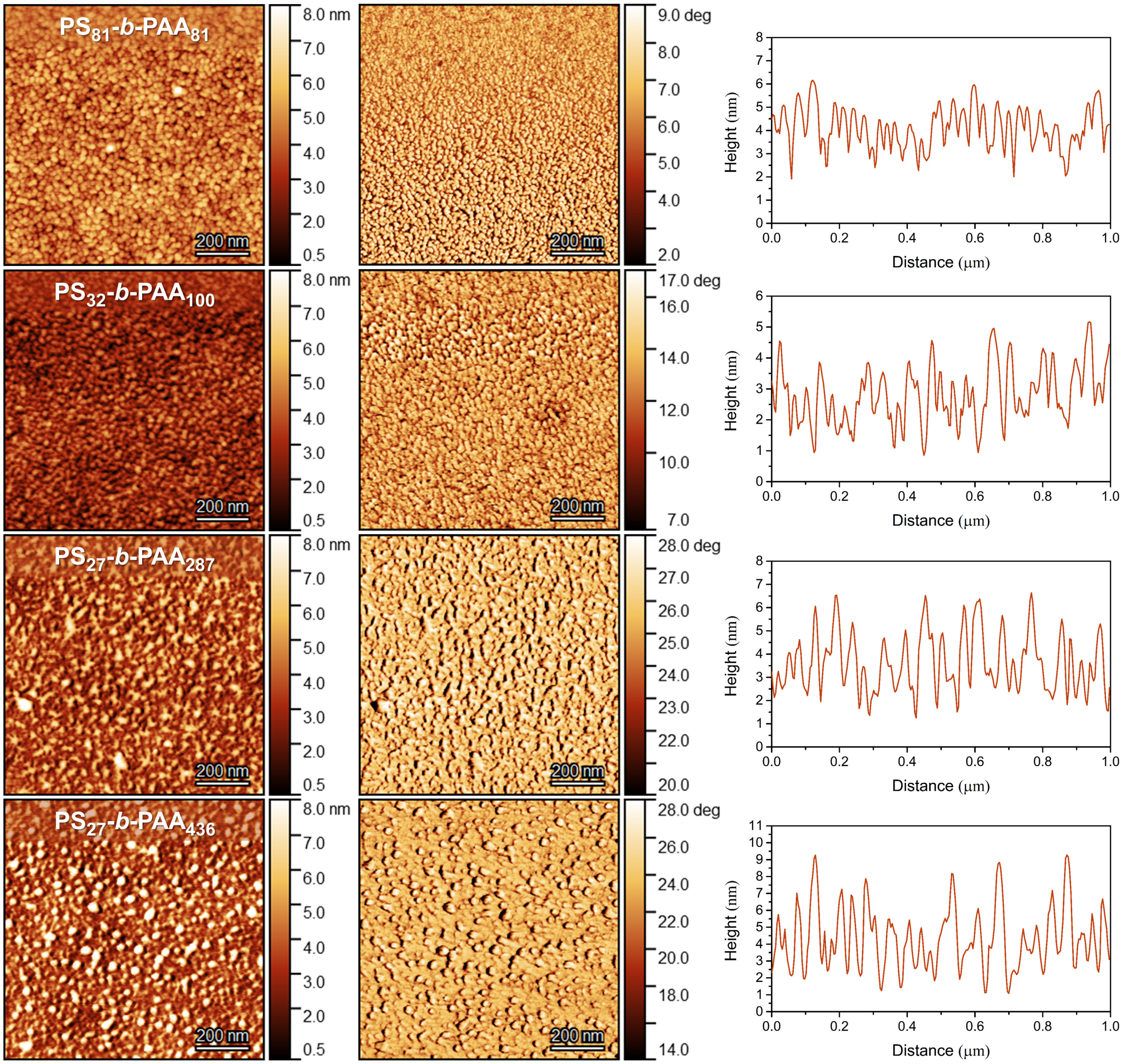

Figure S14. Tapping mode AFM height images (left), phase images (middle), and cross-sectional profiles (right) of the adsorbed PS-*b*-PAA films with various block ratios and lengths.

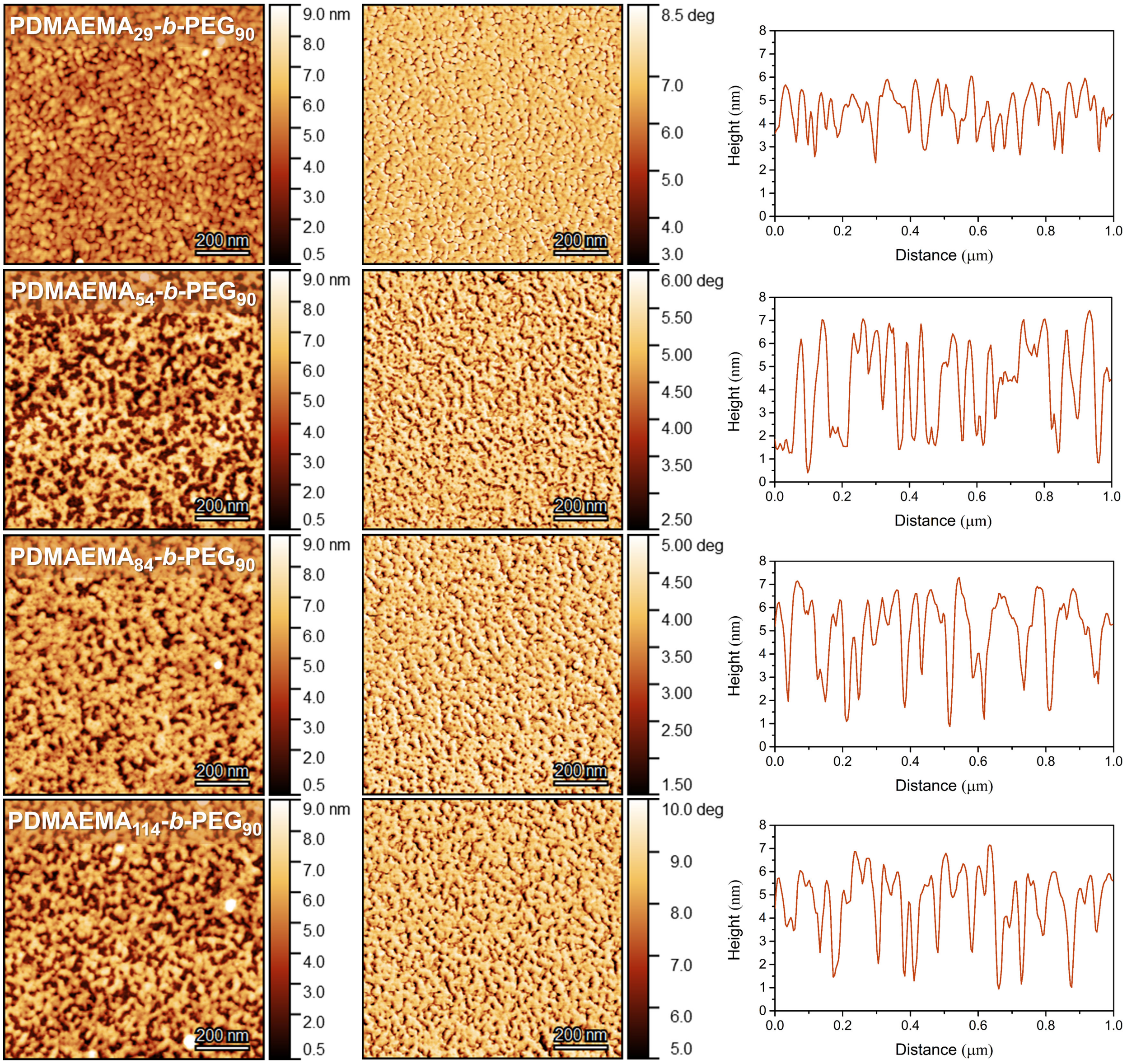

Figure S15. Tapping mode AFM height images (left), phase images (middle), and cross-sectional profiles (right) of the adsorbed PEG-based zipper brush coatings.

Table S7. Summarized characteristics of the adsorbed PEG-based zipper brush coatings, including the ratio between the two polyelectrolyte (PE) blocks (PAA/PDMAEMA), the wet thickness calculated from QCM-D (Voigt model), the dry thickness measured by ellipsometry, the calculated grafting density (σ_{brush}) and charge compensation, the RMS surface roughness (S_q) obtained from tapping mode AFM, and the recorded static contact angles (θ) in water. The grafting densities and charge compensation were calculated according to the methods described in Section 2 of the SI. The captured contact angle images are shown in Figure S16 below. Each denoted value represents the mean ± standard deviation calculated based on at least two (ellipsometry), three (CA), or six (AFM) different spots on the coated sensors, while taking into account the precision and accuracy of each analysis method.

Diblock Copolymer	PE Block Ratio	Wet Thickness (nm)	Dry Thickness (nm)	σ_{brush} (nm^{-2})	Charge Comp.	S_q (nm)	θ (°)
PDMAEMA$_{29}$-*b*-PEG$_{90}$	2.8	18	3.9 (± 0.1)	0.12	0.34	1.1 (± 0.1)	52.4 (± 2.1)
PDMAEMA$_{54}$-*b*-PEG$_{90}$	1.5	19	2.9 (± 0.1)	0.02	0.13	1.8 (± 0.1)	66.5 (± 0.7)
PDMAEMA$_{84}$-*b*-PEG$_{90}$	1.0	18	3.9 (± 0.1)	0.06	0.52	1.6 (± 0.1)	63.3 (± 0.7)
PDMAEMA$_{114}$-*b*-PEG$_{90}$	0.7	15	3.1 (± 0.2)	0.02	0.24	1.7 (± 0.1)	67.5 (± 1.5)

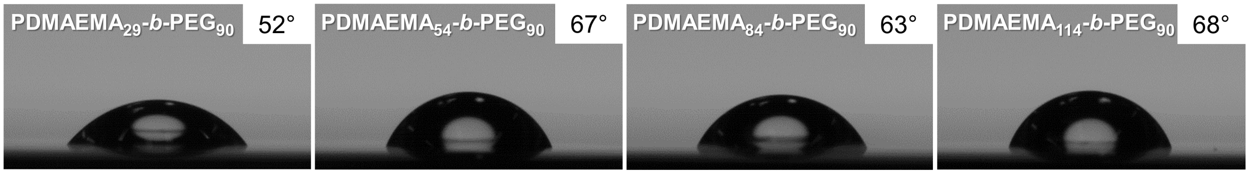

Figure S16. Static contact angles of the adsorbed PEG-based zipper brush coatings, representing the intrinsic water wettability.

Table S8. Summarized characteristics of the adsorbed zipper brush coatings with different antifouling blocks, including the wet thickness calculated from QCM-D (Voigt model), the dry thickness measured by ellipsometry, the calculated grafting density (σ_{brush}), the RMS surface roughness (S_q) obtained from tapping mode AFM, and the recorded static contact angles (θ) in water. The grafting densities were calculated according to the method described in Section 2 of the SI. The captured contact angle images are shown in Figure S17 below. Each denoted value represents the mean ± standard deviation calculated based on at least two (ellipsometry), three (CA), or six (AFM) different spots on the coated sensors, while taking into account the precision and accuracy of each analysis method.

Diblock Copolymer	Wet Thickness (nm)	Dry Thickness (nm)	σ_{brush} (nm^{-2})	S_q (nm)	θ (°)
PDMAEMA$_{29}$-*b*-PEG$_{90}$	18	3.9 (± 0.1)	0.12	1.1 (± 0.1)	52.4 (± 2.1)
PDMAEMA$_{30}$-*b*-POEGMA$_{97}$	19	4.2 (± 0.1)	0.04	1.7 (± 0.1)	60.9 (± 0.5)
PDMAEMA$_{30}$-*b*-PMPC$_{106}$	20	4.5 (± 0.1)	0.04	0.8 (± 0.1)	30.7 (± 0.5)

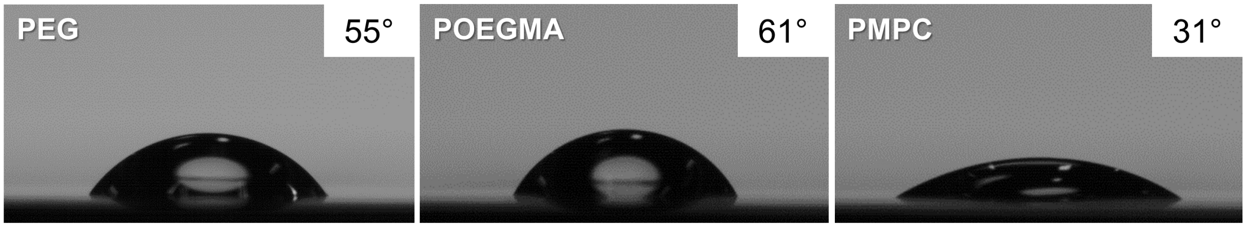

Figure S17. Static contact angles of the adsorbed zipper brush coatings with different antifouling blocks, representing the intrinsic water wettability. The diblock copolymers involved, from left to right: $PDMAEMA_{29}$-*b*-PEG_{90}, $PDMAEMA_{30}$-*b*-$POEGMA_{97}$, and $PDMAEMA_{30}$-*b*-$PMPC_{106}$.

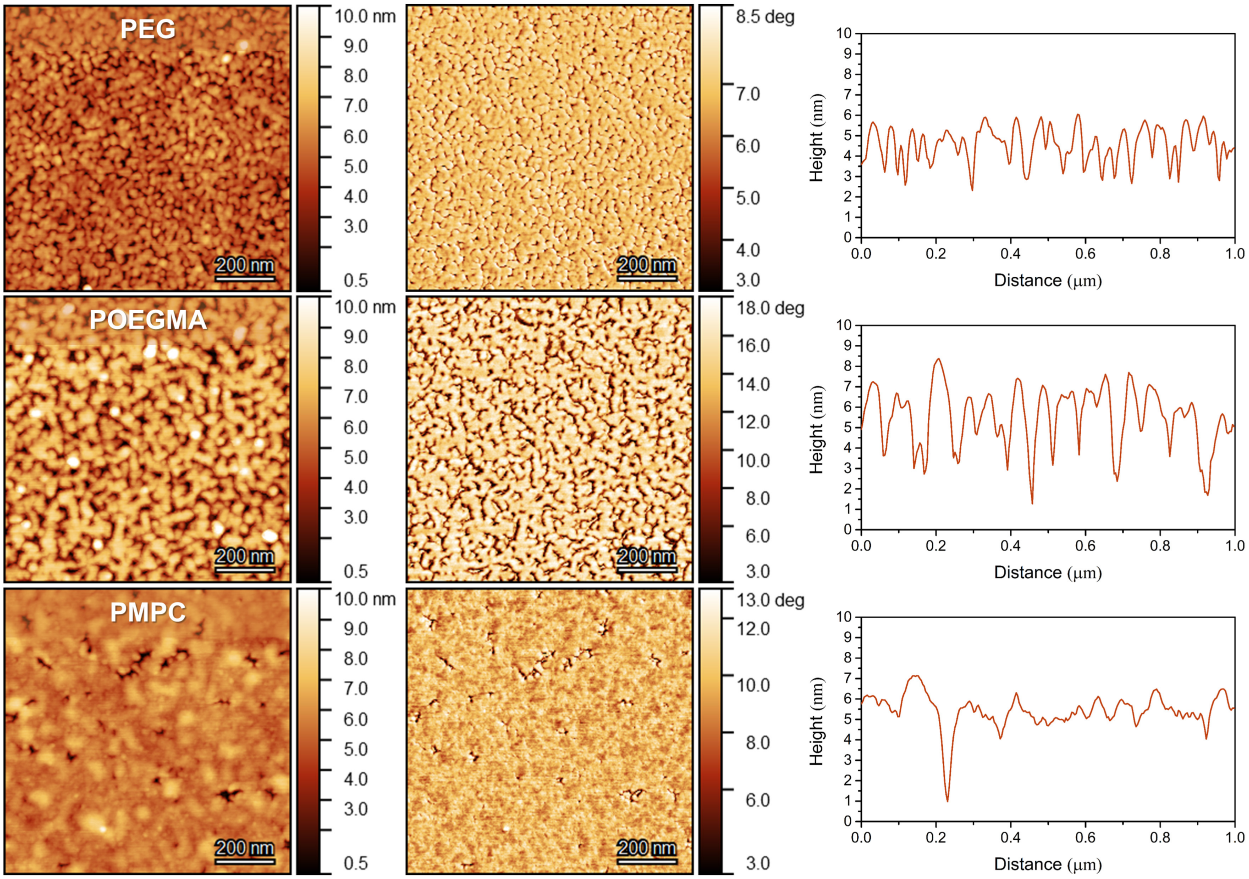

Figure S18. Tapping mode AFM height images (left), phase images (middle), and cross-sectional profiles (right) of the adsorbed zipper brush coatings with different antifouling blocks, involving the specific diblock copolymers $PDMAEMA_{29}$-*b*-PEG_{90}, $PDMAEMA_{30}$-*b*-$POEGMA_{97}$, and $PDMAEMA_{30}$-*b*-$PMPC_{106}$.

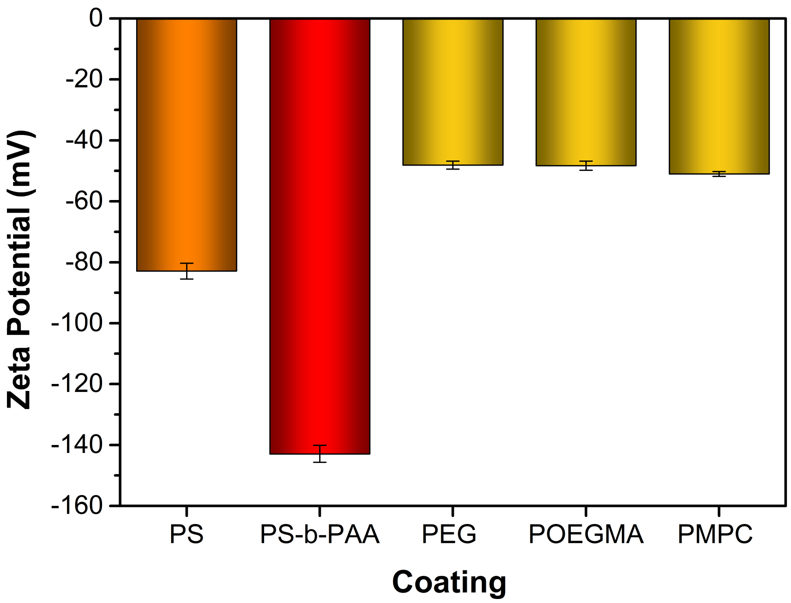

Figure S19. Bar graph summarizing the surface zeta potentials of the PS substrate, PS-*b*-PAA primer, and the adsorbed zipper brushes with different antifouling blocks, measured using the streaming potential technique. The zeta potentials were recorded in a 1 mM KCl electrolyte solution with a pH close to 7. Due to the inaccuracy of the employed technique regarding the investigated coatings, the absolute values should be considered with caution.

References

(1) Hofman, A. H.; Fokkink, R.; Kamperman, M. A Mild and Quantitative Route towards Well-Defined Strong Anionic/Hydrophobic Diblock Copolymers: Synthesis and Aqueous Self-Assembly. *Polym. Chem.* **2019**, *10* (45), 6109–6115.

(2) Pelras, T.; Hofman, A. H.; Germain, L. M. H.; Maan, A. M. C.; Loos, K.; Kamperman, M. Strong Anionic/Charge-Neutral Block Copolymers from Cu(0)-Mediated Reversible Deactivation Radical Polymerization. *Macromolecules* **2022**, *55* (19), 8795–8807.

(3) Obata, M.; Tanaka, S.; Mizukoshi, H.; Ishihara, E.; Takahashi, M.; Hirohara, S. RAFT Synthesis of Polystyrene-Block-Poly(Polyethylene Glycol Monomethyl Ether Acrylate) for Zinc Phthalocyanine-Loaded Polymeric Micelles as Photodynamic Therapy Photosensitizers. *J. Polym. Sci. Part A Polym. Chem.* **2018**, *56* (5), 560–570.

(4) Filippov, A. D.; Van Hees, I. A.; Fokkink, R.; Voets, I. K.; Kamperman, M. Rapid and Quantitative De-Tert-Butylation for Poly(Acrylic Acid) Block Copolymers and Influence on Relaxation of Thermoassociated Transient Networks. *Macromolecules* **2018**, *51* (20), 8316–8323.

(5) Wu, T.; Gong, P.; Szleifer, I.; Vlček, P.; Šubr, V.; Genzer, J. Behavior of Surface-Anchored Poly(Acrylic Acid) Brushes with Grafting Density Gradients on Solid Substrates: 1. Experiment. *Macromolecules* **2007**, *40* (24), 8756–8764.

(6) Davis, K. A.; Matyjaszewski, K. Atom Transfer Radical Polymerization of Tert-Butyl Acrylate and Preparation of Block Copolymers. *Macromolecules* **2000**, *33* (11), 4039–4047.

(7) Thérien-Aubin, H.; Chen, L.; Ober, C. K. Fouling-Resistant Polymer Brush Coatings. *Polymer.* **2011**, *52* (24), 5419–5425.

(8) Michalek, L.; Barner, L.; Barner-Kowollik, C. Polymer on Top: Current Limits and Future Perspectives of Quantitatively Evaluating Surface Grafting. *Adv. Mater.* **2018**, *30* (21), 1–18.

(9) Brandrup, J.; Immergut, E. H.; Grulke, E. A. *Polymer Handbook*, 4th ed.; John Wiley and Sons, Inc., 1999.

(10) Sastri, V. R. Commodity Thermoplastics. *Plast. Med. Devices* **2010**, 73–119.

(11) Hiraoka, K.; Shin, H.; Yokoyama, T. Density Measurements of Poly(Acrylic Acid) Sodium Salts. *Polym. Bull.* **1982**, *8*, 303–309.

(12) Sanjuan, S.; Perrin, P.; Pantoustier, N.; Tran, Y. Synthesis and Swelling Behavior of PH-Responsive Polybase Brushes. *Langmuir* **2007**, *23* (10), 5769–5778.

(13) Li, D.; Sharili, A. S.; Connelly, J.; Gautrot, J. E. Highly Stable RNA Capture by Dense Cationic Polymer Brushes for the Design of Cytocompatible, Serum-Stable SiRNA Delivery Vectors. *Biomacromolecules* **2018**, *19* (2), 606–615.

(14) Bailey, F. E.; Koleske, J. V. Polyoxyalkylenes. *Ullmann's Encycl. Ind. Chem.* **2000**.

(15) Feng, W.; Zhu, S.; Ishihara, K.; Brash, J. L. Protein Resistant Surfaces: Comparison of Acrylate Graft Polymers Bearing Oligo-Ethylene Oxide and Phosphorylcholine Side Chains. *Biointerphases* **2006**, *1* (1), 50–60.

(16) Iwata, R.; Suk-In, P.; Hoven, V. P.; Takahara, A.; Akiyoshi, K.; Iwasaki, Y. Control of Nanobiointerfaces Generated from Well-Defined Biomimetic Polymer Brushes for Protein and Cell Manipulations. *Biomacromolecules* **2004**, *5* (6), 2308–2314.

(17) Kamon, Y.; Kitayama, Y.; Itakura, A. N.; Fukazawa, K.; Ishihara, K.; Takeuchi, T. Synthesis of Grafted Phosphorylcholine Polymer Layers as Specific Recognition Ligands for C-Reactive Protein Focused on Grafting Density and Thickness to Achieve Highly Sensitive Detection. *Phys. Chem. Chem. Phys.* **2015**, *17* (15), 9951–9958.

(18) Brittain, W. J.; Minko, S. A Structural Definition of Polymer Brushes. *J. Polym. Sci. Part A Polym. Chem.* **2007**, *45* (16), 3505–3512.

(19) Rubinson, K. A.; Krueger, S. Poly(Ethylene Glycol)s 2000-8000 in Water May Be Planar: A Small-Angle Neutron Scattering (SANS) Structure Study. *Polymer.* **2009**, *50* (20), 4852–4858.

(20) De Vos, W. M.; Kleijn, J. M.; de Keizer, A.; Cohen Stuart, M. A. Ultradense Polymer Brushes by Adsorption. *Angew. Chemie Int. Ed.* **2009**, *48* (29), 5369–5371.

(21) Seitz, M. E.; Burghardt, W. R.; Shull, K. R. Micelle Morphology and Mechanical Response of Triblock Gels. *Macromolecules* **2009**, *42* (22), 9133–9140.
